# Deep attention networks reveal the rules of collective motion in zebrafish

**DOI:** 10.1371/journal.pcbi.1007354

**Published:** 2019-09-13

**Authors:** Francisco J. H. Heras, Francisco Romero-Ferrero, Robert C. Hinz, Gonzalo G. de Polavieja

**Affiliations:** Champalimaud Research, Champalimaud Centre for the Unknown, Lisbon, Portugal; Radboud Universiteit Nijmegen, NETHERLANDS

## Abstract

A variety of simple models has been proposed to understand the collective motion of animals. These models can be insightful but may lack important elements necessary to predict the motion of each individual in the collective. Adding more detail increases predictability but can make models too complex to be insightful. Here we report that deep attention networks can obtain a model of collective behavior that is simultaneously predictive and insightful thanks to an organization in modules. When using simulated trajectories, the model recovers the ground-truth interaction rule used to generate them, as well as the number of interacting neighbours. For experimental trajectories of large groups of 60-100 zebrafish, *Danio rerio*, the model obtains that interactions between pairs can approximately be described as repulsive, attractive or as alignment, but only when moving slowly. At high velocities, interactions correspond only to alignment or alignment mixed with repulsion at close distances. The model also shows that each zebrafish decides where to move by aggregating information from the group as a weighted average over neighbours. Weights are higher for neighbours that are close, in a collision path or moving faster in frontal and lateral locations. The network also extracts that the number of interacting individuals is dynamical and typically in the range 8–22, with 1–10 more important ones. Our results suggest that each animal decides by dynamically selecting information from the collective.

## Author summary

Simple models have traditionally been very successful, because they usually provide more insight than complicated models. This is particularly true in physics, where simple models can often give highly precise quantitative predictions. However, biology is fundamentally complex and thus it is difficult to find simple models that give precise predictions. To create models that are both precise and insightful, we propose to harness the power of deep neural networks but to confine them into modules with a low number of inputs and outputs. We trained one such model to predict the future turning side of a fish in a collective. By plotting the different modules we obtain insight about how fish interact and how they aggregate information from different neighbours. This aggregation is dynamical and shows that fish can interact with approximately 20 neighbours but can also focus on fewer neighbours, down to 1-2, when some move at higher speed in front or to the sides, are very close or are in a collision path.

## Introduction

There is a wide range of models of collective behavior. A useful way to understand the relative merits of these models is to classify them by their accuracy and their complexity (e.g. [1, 2]). Some of the classical models of collective behavior, like interaction models [3–7], many-eyes or weighted averages [8–11], Condorcet [12] or others [13–17] are of very low complexity. Low complexity can be formally characterised [18], but in practical terms we can define it as the number of parameters in the model. If a model has a low parameter-complexity, we can write down the mathematical description and study it in detail, leading to an intuitive grasp of the problem and therefore a better design of new experiments. In general, however, this simplicity likely misses important biological components. For this reason, these low-parameter-complexity models are not typically tested in their detailed quantitative predictions, using simpler global parameters instead (but see [19, 20]).

New techniques allow to individually track each animal in large collectives with high precision [21], and can provide enough data to build accurate models of collective behaviour. However, it is difficult to increase accuracy without increasing complexity. For example, we can use the trajectories to train a very precise model based on deep neural networks, because they contain thousands or millions of parameters that can be adjusted to approximate any possible function [22]. Unfortunately, this parameter-complexity typically makes deep neural networks black boxes difficult to analyse. However, a function that is parameter-rich but has few inputs and outputs (i.e. low variable-complexity) can in principle be understood through graphical plotting. Here we propose to use deep attention networks [23–25], because they express the social interaction as a combination of two deep-network modules of few inputs and outputs each, and thus they allow to simultaneously achieve insight and predictive accuracy.

We can illustrate the reduction of variables in a modular model by comparing it against an equivalent non-modular model. Without a modular approach, a case with, say, 25 neighbours, and with each animal being sensitive to the 2D position, speed and orientation of each, would need to be modelled using 25 × 6 = 150 variables for each individual. Studying a problem in 150 dimensions is impractical as insight is very difficult to extract. However, deep attention networks are organized in modules, and for some problems each module might depend only on a small number of variables, of the order of 4–6 in our problem. This reduction from 150 variables to modules of 4–6 variables allows for insight into the rules by analysis of these modules, while still achieving high prediction accuracy.

In addition to requiring high prediction accuracy and insight into the underlying interaction rule, we also require that the network model recovers the interaction rule and number of interacting neighbours in ground-truth data generated by agents following some mathematical rules. We found deep attention networks to be well suited for this recovery, as tested for a variety of interaction rules and different number of interacting neighbours.

## Results

### Predicting the future using a deep interaction network

**Fig 1:**
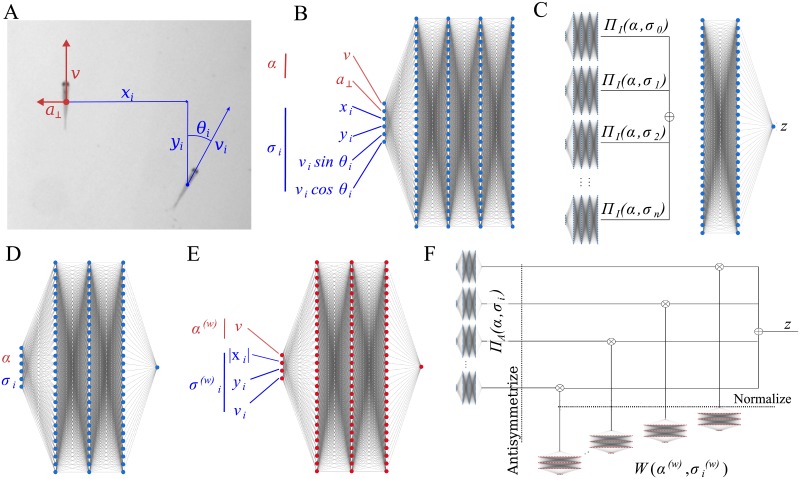
Deep-learning a model of collective behaviour. (**A**) Variables used to predict future turns. Asocial variables, those only involving the focal, in red. Social variables, those involving both the focal and a neighbour, in blue. (**B**) Pair-interaction subnetwork, receiving asocial variables *α* and social variables *σ*_*i*_ from a single neighbour *i*, and outputting a vector of 128 components. All pair-interaction networks share the same weights. (**C**) Interaction network, showing how the outputs of the pair-interaction subnetworks, one for each neighbour, are summed and then fed to an interaction subnetwork. The output, *z* is the logit of the focal fish turning right after 1 s. (**D**) Pair-interaction subnetwork of the attention network. (**E**) Aggregation subnetwork of the attention network. Same structure as D, but the input is a restricted symmetric subset of the variables and the output is passed through an exponential function to make it positive. (**F**) Attention network, showing how the inputs of the pair-interaction and aggregation subnetworks are integrated to produce a single logit *z* for the focal fish turning right after 1 s.

We recorded videos of groups of 60, 80 or 100 juvenile zebrafish, *Danio rerio*, (Fig 1A for a detail; [21] for setup). We tracked videos using our system idtracker.ai, obtaining high-quality position, velocity and acceleration values (see Methods and materials). See also S1, S2 and S3 Figs for statistics analysis of the trajectories obtained for videos of 100, 80 and 60 individuals, respectively, including distance to center of arena, local polarization, interindividual distance and probability of finding another animal around a focal one.

We used the trajectories to obtain data-driven models of fish interactions. First, we required our models to be predictive of the future of a focal fish in test data (video sequences not used to train the model). The requirement of biological insight, which we discuss in the next section, was only added later. The reason for this strategy is that we first need to find out how much we can predict from video and models designed to be insightful need to make assumptions that may reduce the ability to predict.

We used a deep interaction network, inspired by their success in interacting systems in Physics [26]. Our deep interaction network is divided in two parts: (i) *n* pair-interaction subnetworks, each describing the interaction of a focal fish with one of its *n* closest neighbors (Fig 1B), and (ii) an aggregation or weighting subnetwork, aggregating the *n* outputs of the pair-interaction subnetworks (Fig 1C, subnetwork to the right).

The inputs to the network are quantities expressed in a coordinate system centered at the focal fish and with the *y*-axis in the direction of the velocity of the focal (Fig 1A, red). The pair-interaction subnetwork has as inputs the asocial information of the focal, *α* (Fig 1B, red), and the social information of one neighbour *i*, *σ*_*i*_ (Fig 1B, blue). The asocial information of the focal is its speed, *v*, tangential acceleration, *a*_∥_, and normal acceleration, *a*_⊥_. We found that *a*_∥_ had little impact on accuracy (S1 Table), so we did not consider it in further computations. The social information is the neighbour position with respect to the focal, *x*_*i*_ and *y*_*i*_, its velocity, *v*_*i*,*x*_ and *v*_*i*,*y*_, and acceleration, *a*_*i*,*x*_ and *a*_*i*,*y*_. Neighbour accelerations had little impact on accuracy (S1 Table) and were not used in further computations.

Accuracy of prediction of the turning side of the focal fish after 1 second evaluated on held-out test data improves with the number of neighbours, but with diminishing returns (S4 Fig); we chose *n* = 25 neighbors. In the main text we provide analysis of groups of 100 animals and prediction at 1 s in the future for illustration purposes. Our models predict well a range of futures (S5 Fig). Results on how fish interact were found to be similar in computations using 250 ms, 500 ms and 1.5 s in the future (S6, S7 and S8 Figs) and for groups of 60 or 80 zebrafish (S9 and S10 Figs).

Accuracy of prediction of the turning side after 1 s is higher for large turning angles than for turning angles close to 0 or 180 degrees (S11 Fig). For turning angles of 20–160°, the interaction network predicted the correct side with an accuracy of 85.6%; up to 86.8% for 30–100°. In contrast, a model using only focal variables failed to obtain a high accuracy and reached only 60%. Interaction networks with different architectures performed slightly worse (S2 Table), while fully-connected networks performed consistently worse (S3 Table).

The high accuracy of the interaction network shows that the 6 × 25 = 150 dimensions capture an important part of the collective dynamics. The instances not predicted may originate from a variety of effects, including higher-order correlations, individuality and non-markovian effects, i.e. history-dependency, be it at short scales or at long scales (internal states or unaccounted behavioural variables, like posture or eye movements). For instance, adding a history of previous locations of focal and neighbour fish improved accuracy (S12 Fig).

### Deep attention networks obtain a predictive and analyzable model

The deep interaction model of the previous section is too high-dimensional to provide useful insight into animal interactions. The pair interaction subnetwork, for example, takes the values of 6 variables as inputs and outputs 128 values (Fig 1B). The aggregation subnetwork first sums up the 25 128-dimensional vectors to give a single vector of 128 components, and then processes it to output a single number, *z* (Fig 1C).

To gain insight on the nature of fish interactions, we will add hypotheses into the structure of the solution. Specifically, like others before us (e.g. [27–29]), we will assume that animal interactions take place in animal pairs and that each animal integrates with weights these pair interactions,

$$\begin{array}{c} \sum_{i=1}^{n}\Pi \left(x_{i}\right)\frac{W(x_{i})}{\sum_{j}W\left(x_{j}\right)}, \end{array}$$ (1)

where Π is the pair-wise interaction function and *W* is the weighting function.

A possible approach would be to assume simple functional forms for these functions and estimate the parameters from error minimization (like e.g. [30]). Here we explore the alternative of using deep neural networks, powerful function approximators [22], to be shaped into the needed functions by minimizing the prediction error. To this end, we will use two network modules, one capturing the interaction between pairs of fish (Fig 1D), and a second one capturing the aggregation of pair-interactions (Fig 1E). Using these two modules, we can express the probability that the focal turns to the right after 1 s, *p*, as *p* = 1/1 + exp(−*z*), where *z* is the logit that the network outputs (Fig 1F),

$$\begin{array}{c} z=\sum_{i=1}^{n}\Pi \left(\alpha ,\sigma_{i}\right)\frac{W(\alpha^{\left(w\right)},\sigma_{i}^{\left(w\right)})}{\sum_{j}W\left(\alpha^{\left(w\right)},\sigma_{j}^{\left(w\right)}\right)}. \end{array}$$ (2)

The pair-interaction subnetwork, Π(*α*, *σ*_*i*_), describes the interaction of the focal and one neighbour *i*. The aggregation subnetwork *W* gives different weights to the different neighbors *i* in the aggregation depending on the kinematic parameters of focal *α*^(w)^ and neighbor relative to focal $\sigma_{i}^{\left(w\right)}$. The subscript indicates that these variables are, in general, different to the ones in the pair-interaction subnetwork. We found that focal speed, neighbor location and neighbour speed as inputs to the aggregation subnetwork can make an accurate model (S4 Table), which we will use in main text. An alternative model with the same number of variables considers the neighbour velocity (not simply neighbour speed) but does not need the focal speed (S4 Table). For this second model, consider results in S19 Fig.

Since we want the pair-interaction subnetwork, Π, and the aggregation subnetwork, *W*, to represent the logit of turning after 1 s given a neighbor and a weight representing the importance of that neighbor, respectively, they must differ on several accounts. (i) Π can have any real output, while *W* must be always positive. (ii) Π must be antisymmetric with respect to reflection on the y-axis, while *W* must be symmetric. This is because we assume (like e.g. [30]) that a neighbour to the right makes the focal go to the right as much as an identical neighbour to the left of the focal makes the focal move to the left. For the aggregation weight *W*, however, we assume that the importance of the two cases is the same. (iii) The aggregation weights must sum 1. These three conditions are required and we enforced them by: (i) using an exponential as final activation function of *W*, (ii) antisymmetrizing Π and using symmetric input in *W*, and (iii) normalising the outputs of *W* by the sum across all neighbours prior to the integration with the outputs of Π.

The network structure given by equation Eq (2) has been called deep attention network [23, 24]. We trained the deep attention network with our trajectories, achieving 85.1-85.3% accuracy for turns between 20° and 160° and around 86.6% for 30-100°. This is slightly less accurate than the interaction network, but the much lower dimensionality of the two subnetworks allows for a detailed analysis. An attention network trained using shuffled neighbour trajectories only reached an accuracy of 70.3% (large turns, 20°–160°), suggesting that the model trained on normal (unshuffled) data is mainly capturing real interactions and relatively few spurious correlations.

### The structure of interaction of a pair of animals in a collective

**Fig 2:**
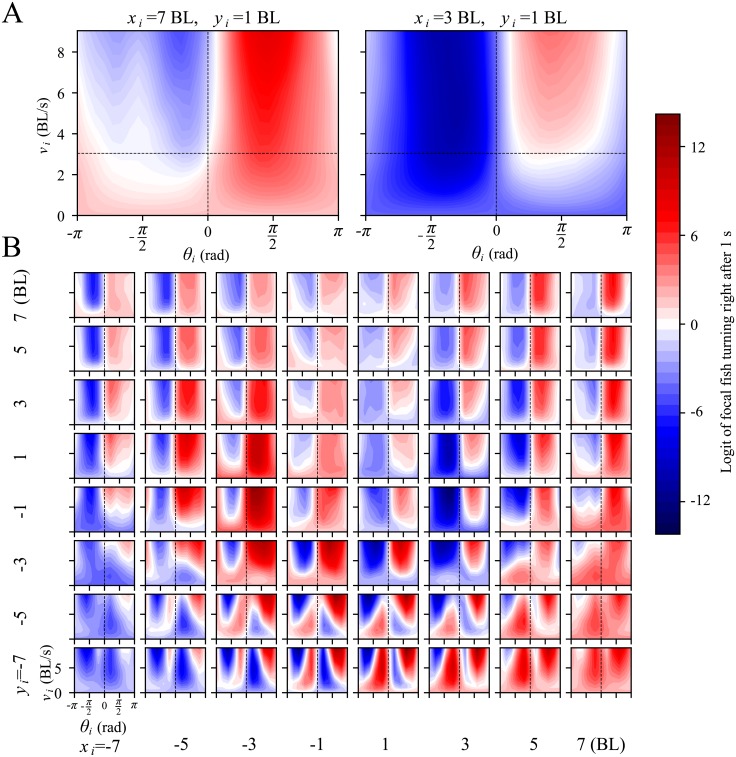
Properties of interaction between a pair of fish in the collective. (**A**) Logit *z* resulting from the pair-interaction subnetwork of the attention network, plotted as a function of the orientation of the neighbour respect to the focal, *θ*_*i*_, and speed of the neighbour, *v*_*i*_, for neighbour located at (*x*_*i*_, *y*_*i*_) = (7, 1) BL (body lengths, left) and (*x*_*i*_, *y*_*i*_) = (3, 1) BL (right). Focal speed is fixed at median speed of 3.04 BL/s and focal acceleration at *a*_⊥_ = 0 BL/s^2^. Red colour is evidence that the focal fish will turn right in 1s, while blue is evidence that the focal fish will turn left. Horizontal dashed line highlights the median speed of 3.04 BL/s. (**B**) Same as (A) but for 64 different neighbour positions (*x*_*i*_, *y*_*i*_), with *x*_*i*_ and *y*_*i*_ taking values in (−7, −5, −3, −1, 1, 3, 5, 7) BL.

The pair-interaction subnetwork Π is a six-dimensional function. We plotted its output, the logit of the focal fish turning to the right after 1 s, *z*, as a function of two variables: the angle, *θ*_*i*_, and the speed of the neighbour, *v*_*i*_ (Fig 2). We fixed the other four variables: focal at median speed of 3.04 BL/s, focal normal acceleration at *a*_⊥_ = 0, and neighbour position at *x*_*i*_ = 7 BL and *y*_*i*_ = 1 BL. At a neighbour speed above the median (Fig 2A, left; median speed indicated with a horizontal line at 3.04 BL/s), the focal animal is sensitive to the neighbour orientation, with a high probability of turning right (left) after 1 s when the neighbour is moving away from (towards) the focal, resulting in an alignment of the focal to the neighbour. When the neighbour speed is below the median, however, the focal is attracted towards it regardless of the neighbour orientation.

As a contrasting example, consider when the neighbour is closer and slightly in front, at *x*_*i*_ = 3 BL and *y*_*i*_ = 1 BL (Fig 2A, right). In this case, the focal gets repelled by the neighbour when the neighbor speed is below 3 BL/s.

These two examples illustrate how alignment, attraction and repulsion depend not only on the neighbour location but also on its speed (Fig 2B, similar to Fig 2A but for a 8x8 matrix of subplots, each for a different neighbour position), and on the speed (S13, S14, S15 and S16 Figs) and acceleration of the focal (S17 Fig).

**Fig 3:**
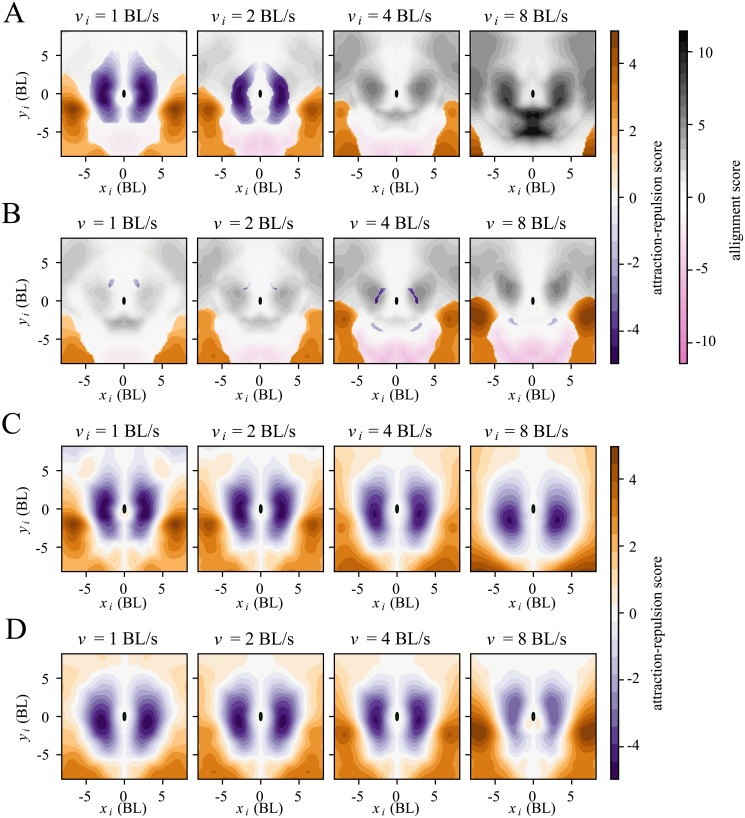
Alignment, attraction and repulsion areas depend on kinematic parameters of focal and neighbour. (**A, B**). Alignment (gray), attraction (orange), repulsion (purple) and anti-alignment (pink) areas. Alignment score (gray) measures how much the logit changes when changing the neighbour orientation angle, and it is computed only in the orientation areas (see Methods and materials). Attraction (orange) and repulsion scores (purple) are the logit averaged across relative orientation angles (positive or negative, respectively), plotted outside orientation areas. (**A**) Scores given at four different values of the neighbour speed (1, 2, 4 and 8 BL/s) while fixing focal speed at the median 3.04 BL/s. Focal normal acceleration fixed at *a*_⊥_ = 0. (**B**) Same as (**A**) but now fixing neighbour speed and varying focal speed. (**C, D**) Attraction and repulsion scores as in (**A,B**) but now plotted for all regions regardless of whether there is alignment effect or not.

From this six-dimensional function we can define alignment regions as those where the logit changes sign with neighbour orientation, that is, when focal will turn right (left) if neighbour orients to the right (left) (Fig 3, gray regions). The alignment score (Eq (12)) measures how sensitive the logit is to neighbour orientation (Fig 3A, gray region; focal speed fixed at median value of 3.04 BL/s and neighbour speed indicated on top of each subplot). The alignment region increases in size and in score with increasing neighbour speed. At high neighbour velocities, strong alignment areas are 2-5 BL behind the focal and 3-5 BL at the sides (Fig 3A, right, darker gray regions). In a region 5-7 BL behind the focal there is a weak orientation effect but reversed in sign, with focal turning right (left) when neighbour orients to the left (right), (Fig 3A, pink). This anti-alignment region extends when increasing focal speed, while keeping neighbour speed fixed at the median value of 3.04 BL/s (Fig 3B, pink).

We define attraction (resp. repulsion) regions as those where the logit does not change sign when changing the neighbour angle. Instead, the focal is attracted towards (resp. repelled away from) the neighbour’s location independently of its orientation. The attraction-repulsion score (Eq (11)) measures how positive (attraction) or negative (repulsion) is the logit of turning towards the neighbour (Fig 3A). Attraction regions shrink with increasing neighbour speed. They are mainly located to the side at 6-8 BL, extending to the back. Repulsion takes place only when the neighbour speed is below the median speed and neighbours are close to the focal (Fig 3A and 3B, purple).

However, classifying interactions into only 4 classes is oversimplistic, and a more complete account is captured by the six dimensional pair-interaction function in Fig 2. For example, when the neighbour is at (*x*_*i*_, *y*_*i*_) = (3, 1) BL and at high speed there is alignment but with a much higher probability of turning left at angles below *π*/2 than turning right at angles above *π*/2. This asymmetry in angles makes the sensitivity to orientation to the neighbour a mix of alignment and repulsion. We can see the full extent of relative attraction and repulsion areas by plotting the attraction-repulsion score for all points in space regardless of whether they correspond to an alignment effect or not (Fig 3C for different neighbour speeds and Fig 3D for different focal speeds). There is an approximately 5 BL diameter region of relative repulsion around the focal. Regions with a mix of alignment and repulsion (or attraction) are those of alignment in Fig 3A and 3B that overlap with regions of relative repulsion (attraction) in Fig 3C and 3D.

We also tested which interactions we would obtain when using shuffled trajectories. We could not find clear repulsion, orientation and attraction areas (S18 Fig), and the outputs are closer to zero. The only exception is the anti-alignment area appearing 5-7 BL behind the focal fish, (Fig 3A and 3B; S18 Fig, pink), consistent with the idea that the anti-alignment areas have an origin in spurious correlation from wall interactions rather than a causal rule of collective motion. This area of anti-alignment corresponds to the complex patterns observed at 5-7 BL behind the focal fish in Fig 2B.

### Recovering interactions in ground-truth data

**Fig 4:**
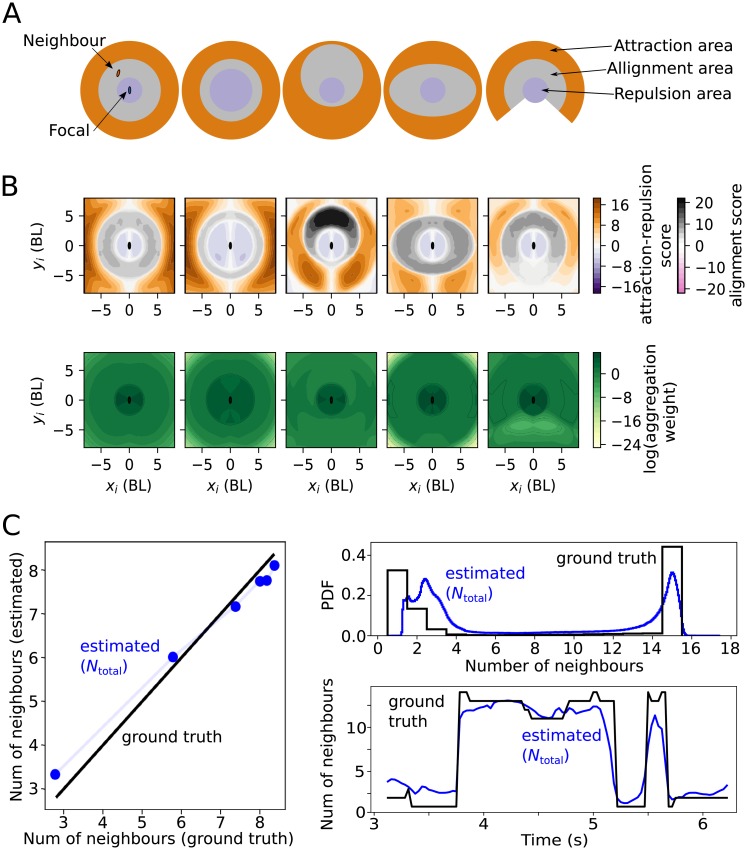
Ground-truth validation using simulated trajectories with known interaction rules. (**A**) Interaction models used to generate data. Fish turn away from neighbours that are in the repulsion area. If there are no neighbours in the repulsion area, fish align with neighbours in the alignment area and are attracted to neighbours in the attraction area. The shape, size and relative location of the areas is varied in different simulations, shown here in different columns. (**B**) Pair interaction scores (above) and aggregation weights (below) obtained when training using simulated trajectories generated by the interaction rules in A. (**C**) Average total number of interacting neighbours, *N*_total_, (blue) as estimated from the deep attention network. Each dot corresponds to a different video with different maximum number of interacting individuals of 3, 7, 11, 15, 19 and 23. Exact correspondence with ground truth number of interacting neighbours as black line. Right: histogram (upper) and example time series (lower) for the estimated number of interacting neighbours, *N*_total_, (blue) and the groundtruth (black). Histogram and time series calculated for trajectories where a maximum of 15 individuals interact.

To validate our ability to reconstruct rules of collective behaviour, we applied our method to simulated trajectories. We generated artificial trajectories of several interacting agents following a variation of well studied behavioural rules [4, 31]. The behavioural rule divides the surroundings of the fish in three areas (Fig 4A, left). If there are neighbours in the innermost area, the fish will turn away from them regardless of the other neighbours. Otherwise, the fish will align with respect to fish in the middle area and turn towards fish in the outermost area (see Methods and materials for details).

Our method successfully recovers the three areas, both correctly labelling them with our definitions of repulsion, orientation and attraction, and matching their spatial extent (Fig 4B, 1st column). We further tested whether we could recover different interaction rules (Fig 4A). We recovered the rule when changing the value of radius of repulsion (Fig 4B, 2nd column), when the orientation area is displaced to the front of the focal (3rd column), when making the orientation area elliptical (4th column) or when adding a blind angle (5th column).

### The aggregation module

The aggregation subnetwork *W* in Eq (2) outputs the (positive) weight of each neighbor in the aggregation. We found it to depend mainly on 4 variables S4 Table

$$\begin{array}{c} W_{i}=W\left(v,v_{i},x_{i},y_{i}\right), \end{array}$$ (3)

with *v* the focal speed, *v*_*i*_ the neighbor speed and *x*_*i*_ and *y*_*i*_ the relative position of neighbour *i*.

**Fig 5:**
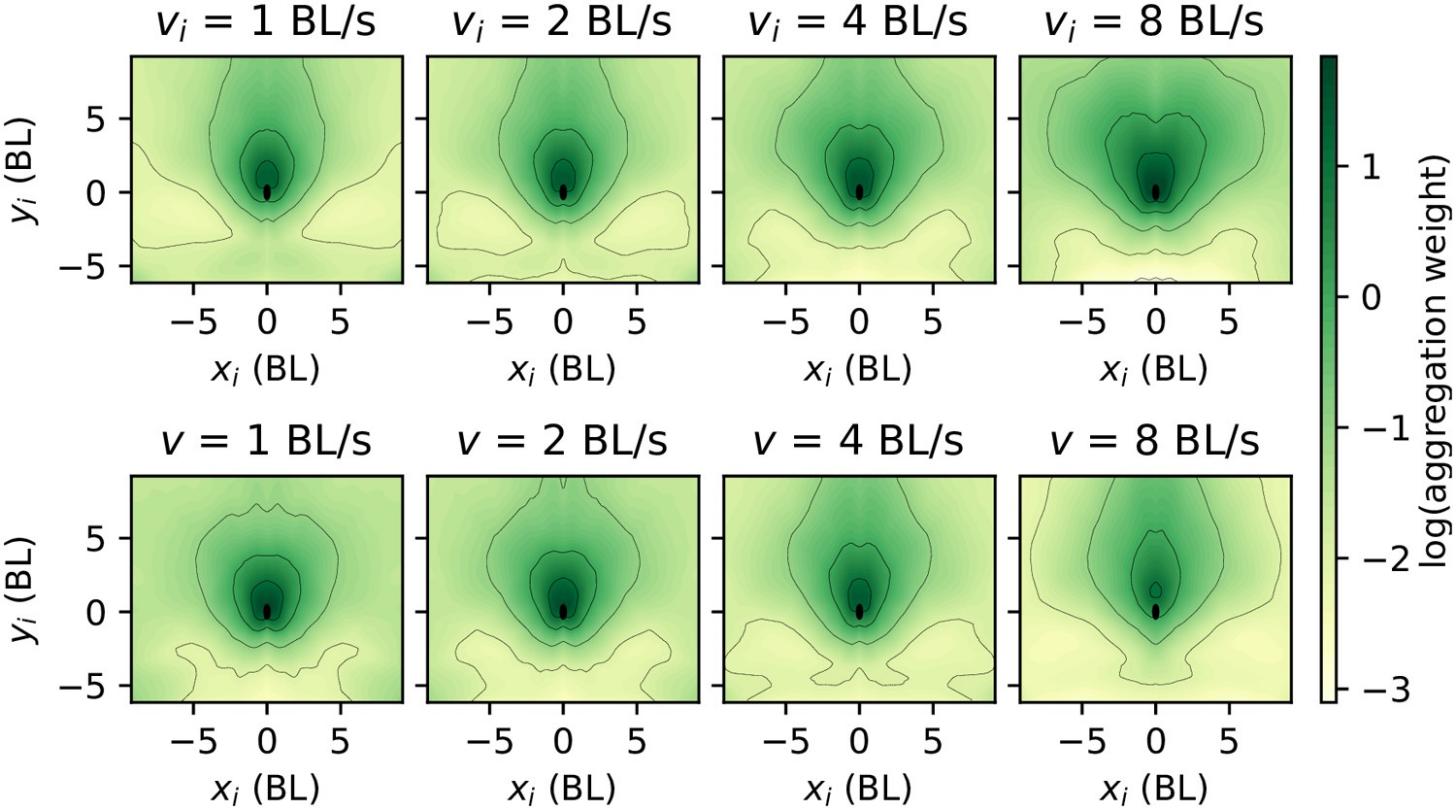
Weighting function: How a fish aggregates information from neighbours. Logarithm of the aggregation weight, log(*W*), as a function of neighbour position, *x*_*i*_ and *y*_*i*_. Top row: focal speed fixed at 3.04 BL/s and each subplot corresponding to different neighbour speeds marked on top of each. Bottom row: same as top row but for fixed neighbour speed at 3.04 BL/s and different focal speeds.

In each subplot of Fig 5 we give *W* for different neighbour positions, keeping neighbour and focal speed constant. Generally, *W* is higher for neighbours that are closer to the focal, and lower for neighbours behind the focal. In contrast, when shuffled trajectories are used during training, the resulting aggregation module shows no clear structure, except for a weak front-back gradient (S18 Fig).

In the upper row of Fig 5 all subplots have the same focal speed at the median speed of 3.04 BL/s, and each indicates the neighbour speed on top, with values *v*_*i*_ = 1, 2, 4 and 8 BL/s. We see how *W* increases with neighbour speed for most neighbour positions, implying that faster neighbours carry more weight in the aggregation. This is more pronounced when the neighbour is close by and to the side.

In the lower row of Fig 5 all subplots have the same neighbour speed at the median speed of 3.04 BL/s, and each indicates the focal speed on top, with values *v*_*i*_ = 1, 2, 4 and 8 BL/s. We see how the mass of *W* increasingly shifts towards the front the faster the focal fish moves. Adding other variables to the attention marginally improves accuracy S4 Table, and still further insight is gained. If the neighbour orientation angle is added as input, higher values of the weight *W* are obtained in positions leading to an immediate collision (S19 Fig).

The final impact of each neighbour on the probability of the focal turning right can be seen from Eq (2) to be given by a normalized weight, that is, the weight given by *W* relative to the sum of the weights of all individuals,

$$\begin{array}{c} \omega_{i}=\frac{W_{i}}{\sum_{j}W_{j}}. \end{array}$$ (4)

For example, if all 25 neighbours are assigned the same weight by *W*, after normalization all animals weight 1/25 = 0.04, no matter how large or small the value of *W* is. If one of the neighbours has a higher (lower) value of *W*, the importance of the other neighbours decreases (increases).

In the network trained using simulated data, the areas corresponding to repulsion are labelled as having an attention several times higher (Fig 4B). This is exactly as it should be, because the collective rules we used to generate the simulated data dictate that the presence of neighbours in the repulsion area causes the focal fish to ignore all neighbours in the orientation and attraction areas.

### Estimating the number of interacting neighbours

Using the aggregation weights, we tested whether it is possible to estimate the number of interacting neighbours. We generated ground-truth trajectories with different numbers of interacting neighbours. Specifically, we generated simulated trajectories with different topological ranges, that is, with different maximum number of interacting neighbours (3, 7, 11, 15, 19 and 23). For this simulated data, the more accurate network was the one that used the topological index of each neighbour in addition to the dynamical variables selected above (80.6% vs 81.6%, all angles), and we thus used it for the following analysis.

Previous work (e.g. [32]) detect social interaction by looking for correlations between trajectories, analysing time delays to determine the direction of the influence. In our case, it is natural to use the aggregation weights as a proxy of the strength of the influence of each neighbour, and based on them we designed functions to quantify the number of the most influential. More specifically, we used the inverse of the typical weight *N*_total_ = 1/*ω*_t_ = exp(− ∑_*i*_ *ω*_*i*_ log(*ω*_*i*_)) ([33], section 4.4). Using this expression, we recovered the average ground-truth values for all the cases in which we used simulated trajectories (Fig 4C, left).

The recovery of the number of interacting neighbours can be seen in more detail using statistics instead of simply the average number of neighbours. We show for illustration this statistics for simulations with a maximum number of neighbours of 15 (Fig 4C). Note that the ground truth has a peak at 1 neighbour, corresponding to a neighbour in the repulsion region, when the simulated agent ignores other neighbours outside the repulsion area (Fig 4C, right, black). The peak at a 15 neighbours in the ground truth corresponds to the number of neighbours within the interacting areas, with a maximum of 15 (Fig 4C, right, black). The statistics of interacting neighbours estimated by the network model has a good correspondence with the ground truth (Fig 4C, right, blue). Consistent with the statistics, the network also correctly estimates the time variation of the number of interacting neighbours, as illustrated in the example of Fig 4C, bottom.

**Fig 6:**
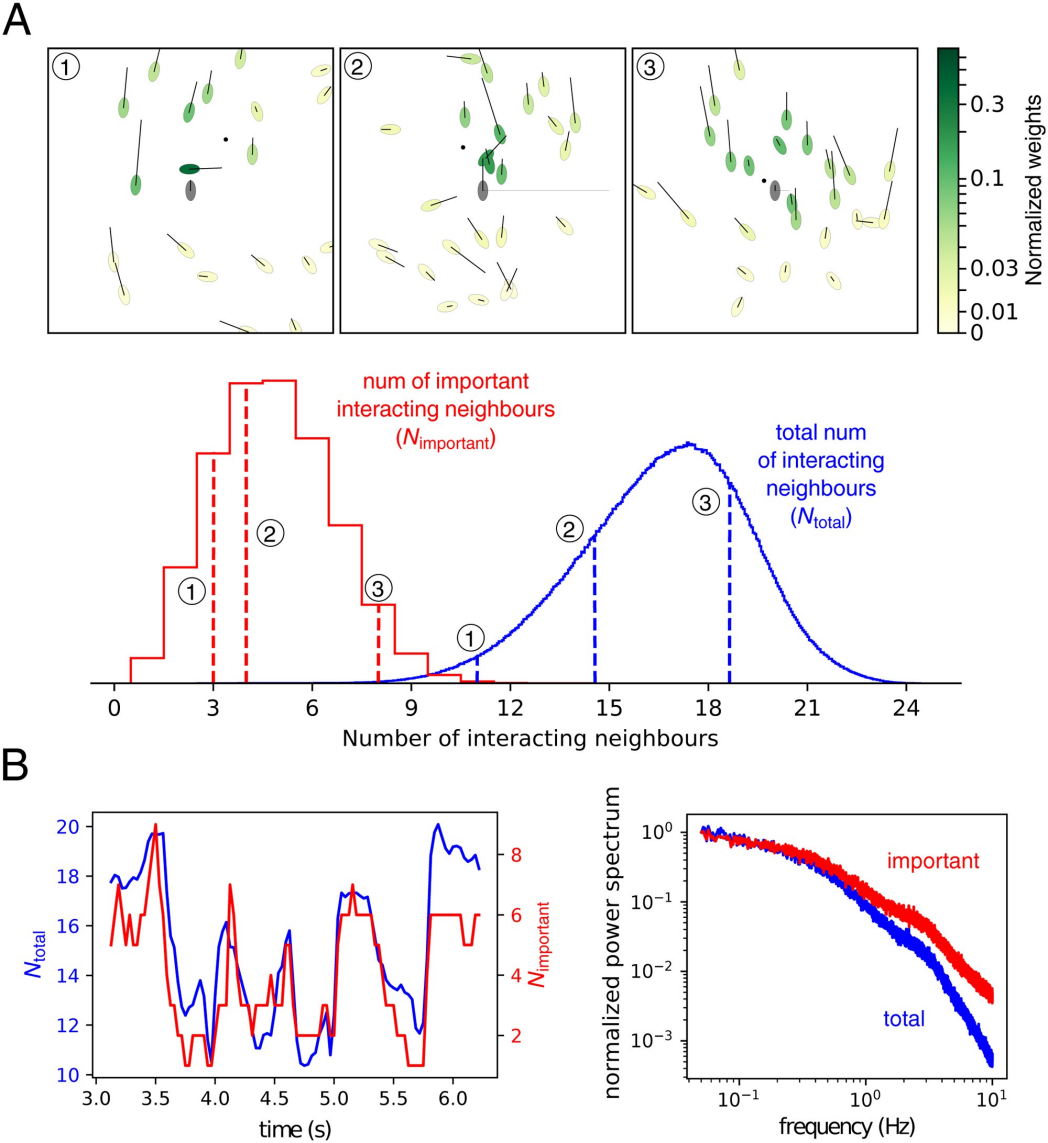
Relevant neighbours in the aggregation. **A** (Upper panel) Three example frames with each neighbour coloured with its normalized weight in the aggregation. Focal animal is indicated in gray color, with a horizontal line proportional to the normal acceleration to either left or right and a small dot in its frontal positions indicating the focal position 1 second into the future. In both focal and neighbours, a line along the major axis indicates the fish velocity. (Lower panel) Distribution of the total number of interacting neighbours *N*_total_ (blue), and of the important neighbours, *N*_important_ (red). **B** Left: Time variation of the number of total interacting neighbours, *N*_total_, and the number of important neighbours, *N*_important_ for a illustrative focal fish and short period of time. Right: Power spectra of the two measures of the estimated number of neighbours.

We performed a similar analysis in the fish experimental data. To illustrate the effect of aggregation in the data, we give the normalized weights *ω*_*i*_ in Eq (4) for each neighbour in three illustrative frames (Fig 6A; animals with higher normalized weights in darker green). We observe cases in which a large number of neighbours has an important impact (Fig 6A, upper right), others with fewer (Fig 6A, upper middle) or even with a single neighbour that overweights the others by far (Fig 6A, upper left).

To quantify this smaller set of important neighbours, we computed the number of neighbours with weight smaller than the typical weight, *N*_important_. For the videos with 100 individuals, the resulting distribution of estimated total number of interacting neighbours *N*_total_ has a mean of 16.6, and the distribution of number of important neighbours *N*_important_ has a mean of 4.8, (Fig 6A, red).

The number of interacting and important neighbours changes dynamically. To gain some intuition on the dynamics of the number of total and important interacting neighbours, consider how these values change in time in an illustrative section of video for a focal individual (Fig 6B, left). It can be seen that both measures of interacting and important neighbours are roughly proportional and that they can change in a fast sub-second scale. We analyzed the time scales by computing the power spectrum of both measures (Fig 6B, right). The power spectra monotonically decrease with frequency, implying that most of the variation occurs at low frequencies, albeit without a concrete value for the time-scale (Fig 6B, right).

## Discussion

We developed a model of collective motion that (i) accurately predicts future behaviour (like e.g. [20, 25, 34, 35]) (ii) is insightful, (iii) works for data not used to obtain the model, a standard requirement in machine learning, and (iv) it recovers the interaction rule and number of neighbours in groundtruth data.

We divided the interaction rule into two functions, a pair-wise interaction function and a second function giving the weight of each neighbour in the aggregation. Instead of fitting a simple functional expression (e.g. [30]) or a shallow network [36], we used a deep neural network, specifically a deep attention network. After training, we found that we could extract attraction, repulsion and alignment as approximate notions [4, 6, 31] from the trained pairwise interaction. Usually, these interaction classes are defined only in terms of relative position of neighbour. However, we found them to exist in a 6-dimensional space. This translates into these classes also depending on speeds of focal and neighbour, focal acceleration and relative orientation between the two fish. This implies that experiments testing for the relevance of one variable, say speed, may give contradictory results depending on the analysis strategy. Our results imply that analysis needs to take into account that the interactions take place in a space with more dimensions. Also note that the three classes are not cleanly separated as alignment regions are mixed with attraction or repulsion, as found in [30].

To calculate the weight in the aggregation we also chose a neural network instead of a simple rule. The neural network architecture we chose has enough flexibility to approximate many previously proposed weighting rules. This includes the angle subtended by the neighbour fish [29], the inverse of the distance to the neighbour [28], and a decreasing function of the topological rank [27]. Other previously proposed rules are binary weights based on thresholds of simulated visual motion cues [37, 38], first Voronoi neighbourhood [7] and topological ranges [39]. A simple aggregation rule would be to average the outputs of the pairwise-interaction network. Compatible with previous studies (e.g. [40]), we found that this simple rule gives a lower accuracy (S4 Table).

Providing it is large enough to be flexible and small enough not to seriously suffer from overfitting, the exact architecture of the two neural subnetworks does not have a strong effect in the obtained maps (S20 and S21 Figs).

An aggregation rule obtained by weighting using functions of dynamical variables can explain data that seem to be generated by a fixed number of neighbours in the interaction, as shown by using a simpler alternative [28]. Our aggregation may be seen as allowing a smooth transition from average type models [3–7, 20, 41] and models in which one or very few animals influence the rest as in the many-eyes model for predator detection [8–11, 42] and others [17]. Note that this ability to shift from many to few can allow a collective to match the changing knowledge distribution in the group [43]. We believe our methodology could open the door to the experimental study of the properties of this matching.

In addition to the attention network, we developed a more precise deep interaction network that taught us which are the relevant variables to consider and gave us a reference accuracy. The interaction network is a smooth interpolation of the raw data, and it may also be used to complement data analysis studies of experimental data. This is particularly true in high-dimensional systems, where data-analysis runs into many practical difficulties. Having an interpolating function that can be queried simplifies the exploration of the space of inputs. Important input variables can be identified, and their effects readily tested for a few different combinations of the other variables. Intuitions obtained from exploring the interpolating function can then be used to produce a model or to guide data analysis. The deep interaction network can also be used for problems requiring prediction of the future location of an animal, for example the optogenetic brain stimulation [44] or microscopic imaging [45] of free-swimming animals.

The variables selected as input to the models can be enriched at the cost of increasing the model dimensionality. For example, one can add more information about behavioral history, possible internal variables (parametrized, for example, by time of day or more direct internal measurements), explicit dynamics and posture [46–49]. For instance, a longer history of previous locations allows the attention network to increase its predicting accuracy, albeit with diminishing returns (S12 Fig). Also, the existence of higher-order interactions not captured by the deep attention network is evidenced by its lower accuracy compared to the interaction network, (S5 Table).

Our results illustrate how modular deep networks enable flexible data-driven modelling without losing insight. Each module is flexible, with tens of thousands of parameters, but implements a function with low dimensionality in the number of inputs and outputs. Combinations of modules [50, 51], two in the attention network we used, can achieve higher compositional complexity that adds flexibility without losing insight.

## Methods and materials

### Data and code availability

60- and 100-fish as well as the new 80-fish videos can be found at https://idtracker.ai. Code used in this study is free and open-source and may be used to study interactions in any animal species or other agents (https://gitlab.com/polavieja_lab/fishandra).

### Ethics statement

We performed the experiments following the approved animal research protocol entitled ‘Decision-making in animal groups: a multidisciplinary approach to understand how social information is processed’ (CF internal reference 2015/007, DGAV reference 0421/000/0002016). The animal research protocol was approved by the Animal Welfare Body of the Champalimaud Foundation (ORBEA FC) and the Portuguese competent authority for animal welfare issues, Direcao Geral de Alimentao e Veterinaria (DGAV).

### Animal rearing and handling

Zebrafish, *D. rerio*, of the wild-type TU strain were raised by the Champalimaud Foundation Fish Platform, according to methods in [52]. Handling procedures were as in [21]. We used juveniles of 31-33 days post fertilization (a body length of 16 mm, subtending ∼ 80 px in the videos).

### Videos and tracking

We used 6 10-minute videos of 60 and 100 freely swimming juvenile zebrafish from [21], and 3 new 10-minute videos of 80 juveniles. The camera had a frame rate of 32 fps and 20 Mpx of definition. We obtained all the fish trajectories using *idtracker.ai* with an accuracy of 99.95 (mean) ±0.01% (std) [21].

### Preprocessing

We interpolated linearly the very small holes in the tracked trajectories (0.027% for 100-fish videos). We normalized trajectories, by translation (center of arena to (0,0)) and scaling (radius of the arena to 1). To reduce noise while preventing contamination by any future information, we smoothed the trajectories using a 5-frame half-Gaussian kernel with *σ* = 1 frame. We obtained velocity and acceleration by finite differences, using only current and past frames. To avoid direct border effects, we removed datapoints where the focal fish is further away from the center than 80% of the radius. Each video was divided in three parts, to obtain the training, validation and test datasets (97%/2%/1%). Sometimes we use one such division, and often we use three with validation and test covering different parts of the video.

In each video frame, for each individual, we found the *n* nearest neighbours (I). We then obtained (i) velocity and acceleration of the focal fish, (ii) relative position, absolute velocity and absolute acceleration of the closest *n* neighbours, (iii) whether the focal fish has turned right or left after *N*_*f*_ frames in the future.

Shuffled trajectories were obtained by shifting in time the trajectories of each individual, before finding the *n* closest neighbours. Each individual was shifted by a different multiple of the number of frames in the video divided by the number of individuals. In 10-minute videos of 100 individuals this strategy guarantees a minimum shift of 6 s and an average shift of 150 s. Properties of the focal fish (speed, acceleration) are not affected by this shuffling.

### Deep networks

We implemented the Deep Networks using Keras [53] through its Python API and with TensorFlow backend [54]. We solved the following classification task: Given dynamical properties of a focal fish and its *n* closest neighbours, does the focal fish turn right or left after 1s? Asocial information is the set of speed and normal and tangential acceleration of the focal,

$$\begin{array}{c} \alpha =\{v,a_{⊥},a_{\|}\ldots \}. \end{array}$$ (5)

Social information from a neighbour *i* is its location, velocity and acceleration

$$\begin{array}{c} \sigma_{i}=\left\{x_{i},y_{i},v_{i},\theta_{i},a_{x,i},a_{y,i},\ldots \right\}, \end{array}$$ (6)

whose coordinates we calculate in an instantaneous frame of reference that is not moving, which is centered in the focal fish and whose y-axis is co-lineal with the focal fish velocity. Note that *v*_*i*_ is the absolute speed, while (*x*_*i*_, *y*_*i*_) is the relative position of the neighbour, rotated to the frame of reference. In each network, we first obtain the logits *z*, and then the probabilities by using a logistic function *p* = 1/(1 + *e*^−*z*^).

#### Interaction network

In the interaction network [26], given asocial (*α*) and social ($\left\{\sigma_{i},i\in I\right\}$) information, the logit of turning right is calculated as

$$\begin{array}{c} z=I\left(\alpha ,\left\{\sigma_{i}\right\}\right)=\Gamma (\sum_{i\in I}\Pi_{I}\left(\alpha ,\sigma_{i}\right)). \end{array}$$ (7)

The function Π_I_ is the pair-interaction subnetwork. We modelled it using a fully-connected network with 3 hidden layers of 128 neurons each, plus a readout layer of 128 neurons. There are rectified linear unit (ReLU, [55]) nonlinearities after each hidden layer (but not after the readout). The outputs of Π_I_ for different neighbours are summed together and transformed by a second function, Γ. We modelled Γ as a fully-connected layer with one hidden layer of 128 neurons, plus a one-neuron readout layer. There are ReLU nonlinearities preceding the whole network and after each hidden layer (but not after the one-neuron readout layer).

To effectively multiply available data by *n*, we considered all neighbours to be equal. Equivalently, there is symmetry with respect to exchange of neighbour labels. We did not observe any turning side preference. Therefore, to effectively multiply available data by 2, we forced the network to be antisymmetrical with respect to a reflection along the body axis by antisymmetrization of *I*,

$$\begin{array}{c} z=I\left(\alpha ,\left\{\sigma_{i}\right\}\right)-I\left(\alpha^{*},\left\{\sigma_{i}^{*}\right\}\right), \end{array}$$ (8)

where the star superscript represents a reflection along the longitudinal axis of the body, calculated by switching the sign of all *x* components.

#### Attention network

Eq (2) can be rewritten using a notation that compares directly with Eq (7) as

$$\begin{array}{c} z=A\left(\alpha ,\left\{\sigma_{i}\right\}\right)=\sum_{i\in I}\Pi_{A}\left(\alpha ,\sigma_{i}\right)\frac{W(\alpha ,\sigma_{i})}{\sum_{j}W\left(\alpha ,\sigma_{j}\right)}. \end{array}$$ (9)

The function Π_A_ captures the effect of pairwise interactions. It has the same structure as Π_I_ except that its readout layer has only one neuron, and that we anti-symmetrise it. *W* is an attention layer, weighting the logits of the different neighbours. *W* has the same structure as Π_A_, except that it accepts as input a y-axis-reflection-invariant subset of the asocial and social variables, and that there is an exponential function after the single-neuron readout signal.

#### Loss

Following standard procedures in binary classification, when training the network to estimate the probability *p*_*i*_ of turning right, we minimised the cross-entropy loss, [55]

$$\begin{array}{c} L=-\frac{1}{N_{b}}\sum_{i=1}^{N_{b}}\log\left(p_{i}^{*}\right). \end{array}$$ (10)

summed along *N*_*b*_ data points in the minibatch and where $p_{i}^{*}$ is the probability given by the network to the actual turn. When the network predicts a right turn with probability *p*_*i*_, $p_{i}^{*}=p_{i}$ if the actual turn was to the right, and $p_{i}^{*}=1-p_{i}$ if the actual turn was to the left. We minimise loss using Adam [55]. We stopped training if validation loss did not reach a new minimum for 10 epochs and did increase 25% from the current minimum, or after 100 training epochs. In the attention network, we annealed learning rate from 10^−4^ to 10^−5^, using a batch size of 500. In the interaction network, we annealed learning rate from 5 × 10^−5^ to 10^−5^ and trained with a batch size of 200. Dropout [55] did not improve accuracy.

### Attraction-repulsion and alignment scores

Attraction-repulsion score is obtained as

$$\begin{array}{c} \text{sign}\left(x\right){\left\langle z_{i}\right\rangle }_{\theta_{i}\in \left[-\pi ,\pi \right)}, \end{array}$$ (11)

where 〈〉_*θ*_*i*_∈[−*π*,*π*)_ indicates average over all possible relative orientation angles of the neighbour. There is attraction (repulsion) when the score is positive (negative). Alignment score is obtained as

$$\begin{array}{c} \underset{\theta_{i}\in \left[-\pi ,\pi \right)}{\text{max}}\left\{z_{i}\;\text{sign}\left(\theta_{i}\right)\right\}-\max_{\theta_{i}\in \left[-\pi ,\pi \right)}\left\{-z_{i}\;\text{sign}\left(\theta_{i}\right)\right\}. \end{array}$$ (12)

If the logit *z*_*i*_ changes sign when changing the relative orientation of the neighbour *θ*_*i*_ we consider that point in parameter space to be in an orientation area. Otherwise, it is in an attraction or repulsion area, depending on the sign of the attraction-repulsion score.

### Simulated trajectories

At time step increment of 1/32 s, animals have a probability 2/3 of not changing its velocity. With probability 1/3, the animal changes the direction of its velocity (not its module: speed is constant) following rules explained in [4]. In some simulations we consider a blind angle behind the fish, and the shape, size and relative position of the different areas are changed (as explained in text and Fig 4). The other parameters of the model (as defined in Table 1 in [4]) and the values we chose are: number of individuals (50), turning rate (0.2 rad), speed (3 BL/s) and error (0.2 rad). All simulated agents are confined inside a circular arena of radius 25 BL, and when touching the arena border they reverse the component of the velocity perpendicular to the border. Training with simulated trajectories was performed to predict turning side after 125 ms.

## Supporting information

S1 Fig Summary of the collective behaviour statistics for 3 videos of 10 minutes of 100 juvenile zebrafish. **A** Probability density function (pdf) of the distance to the center of the arena. The black vertical line marks the point 0.8 radius from the center; data to its right is neither used to train nor to evaluate the model to avoid direct border effects. **B** Polarisation, calculated locally in each frame for each focal fish and a different number of its closest neighbours, both for original and shuffled trajectories. **C** pdf of interindividual distances, in each frame for each fish to each of its closest neighbours. **D** Difference between the pdfs of relative locations of the 25 nearest neighbours in original and shuffled trajectories.
(PDF)

S2 Fig Summary of the collective behaviour statistics for 3 videos of 10 minutes of 80 juvenile zebrafish. As in S1 Fig.
(PDF)

S3 Fig Summary of the collective behaviour statistics for 3 videos of 10 minutes of 60 juvenile zebrafish. As in S1 Fig.
(PDF)

S4 Fig Accuracy of prediction of the turning side of the focal fish after 1 second evaluated on a held-out test data of large turns (20°-160°) as a function of number of closest neighbours. One run for each network/condition. Both the interaction network (blue) and the attention network (orange) improve in accuracy with the number of neighbours, and then plateau after approx. 20 neighbours.
(PDF)

S5 Fig Accuracy of prediction of the turning side of the focal fish evaluated on held-out test data of large turns (20°-160°) for different times to prediction. Mean of three runs, taking the test set at different positions of the video. The prediction from an aggregation model with 25 neighbours (blue) and from a model that is blind to any social information (black). Accuracy for immediate futures (less than 100 ms) is high for both models, because of correlations in the acceleration. Then it decreases for both models, but accuracy with 25 neighbours has a broad maximum when predicting futures between 1 and 10 s, and then slowly drops when predicting more distant futures.
(PDF)

S6 Fig Pair interaction and aggregation in 250 ms predictions. **A** Same as Fig 3A. **B** Same as Fig 3B **C** Same as Fig 5. Note how high-attention areas are closer to the focal fish. **D** Same as Fig 5.
(PDF)

S7 Fig Pair interaction and aggregation in 500 ms predictions. **A** Same as Fig 3A. **B** Same as Fig 3B **C** Same as Fig 5. **D** Same as Fig 5.
(PDF)

S8 Fig Pair interaction and aggregation in 1500 ms predictions. **A** Same as Fig 3A. **B** Same as Fig 3B **C** Same as Fig 5. Note how high-attention areas are closer to the front. **D** Same as Fig 5.
(PDF)

S9 Fig Pair interaction and aggregation, obtained from 60-fish videos. **A** Same as Fig 3A. The most conspicuous difference with Fig 3A is the weakening of anti-alignment **B** Same as Fig 3B **C** Same as Fig 5. **D** Same as Fig 5. Note the comparatively weak attention at the back of the focal.
(PDF)

S10 Fig Pair interaction and aggregation, obtained from 80-fish videos. **A** Same as Fig 3A. The most conspicuous difference with Fig 3A is the weakening of anti-alignment **B** Same as Fig 3B **C** Same as Fig 5. **D** Same as Fig 5.
(PDF)

S11 Fig Accuracy of prediction of the turning side of the focal fish after 1 second evaluated on held-out test data, as a function of turning angle. Data from one single run. When using only focal variables (blue) remains low at all turning angles. Both networks integrating information from 25 neighbours, interaction (orange) and attention (green) perform better at turning angles between 40 and 100.
(PDF)

S12 Fig Accuracy of prediction of the turning side of the focal fish evaluated on held-out test data of large turns (20°-160°) when the interaction subnetwork of the aggregation network uses history of previous positions of focal and neighbour fish. Mean of three runs, taking the test set at different positions in the video. Prediction accuracies increase when information from more frames in the past is available to the network.
(PDF)

S13 Fig Properties of interaction between a pair of fish in the collective when the focal fish is moving at low speed (1 BL/s). (PDF)

S14 Fig Properties of interaction between a pair of fish in the collective when the focal fish is moving at low speed (2 BL/s). (PDF)

S15 Fig Properties of interaction between a pair of fish in the collective when the focal fish is moving at low speed (4 BL/s). (PDF)

S16 Fig Properties of interaction between a pair of fish in the collective when the focal fish is moving at low speed (8 BL/s). (PDF)

S17 Fig Properties of interaction between a pair of fish in the collective when the focal fish is in the midst of a right turn. Focal normal acceleration fixed to *a*_⊥_ = 100 BL/s^2^.
(PDF)

S18 Fig Aggregation, attraction-repulsion, and pair interaction maps for groups of 60, 80 and 100 zebrafish. We show results for the network trained with the original data and the data shuffled according to Methods and Materials. Focal and neighbour speeds fixed to the median.
(PDF)

S19 Fig Aggregation with information about relative orientation. Same as Fig 5, but when the aggregation subnetwork is trained with the relative orientation of the neighbour, in addition to the variables used in the main text. **A** The neighbour is parallel (at 0 degrees) to the focal. **B** The neighbour is at 45 degrees (towards the right) with the focal, **C** the neighbour is perpendicular (90 degrees) and pointing to the right of the focal. **D** The neighbour is antiparallel (180 degrees) to the focal.
(PDF)

S20 Fig Aggregation, attraction-repulsion, and pair interaction maps for different architectures of the attention network. We show results for different number of layers in both, the pair-wise interaction subnetwork, and the aggregation subnetwork. Focal and neighbour speeds fixed to the median.
(PDF)

S21 Fig Aggregation, attraction-repulsion, and pair interaction maps for different architectures of the attention network. We show results for different number of neurons in the layers of both, the pair-wise interaction subnetwork, and the aggregation subnetwork. Focal and neighbour speeds fixed to the median.
(PDF)

S1 Table Validation loss and test accuracy of prediction of the turning side of the focal fish after 1 second, changing input variables. 25 neighbours, interaction network, mean of three runs. In all cases, in addition to the variables mentioned in the table, we provide the relative position of the neighbour (*x*_*i*_, *y*_*i*_). Elsewhere in this article, we use *v*, *a*_⊥_, *v*_*i*_, *θ*_*i*_ and (*x*_*i*_, *y*_*i*_), the simplest among the sets of variables with high accuracy. Average of three runs with different train-validation-test splits.
(PNG)

S2 Table Different architectures of interaction networks (number neurons of per layer × number of hidden layers). Best (i.e. lowest validation loss) of at least three runs with different batch sizes, with a constant train-validation-test split.
(PNG)

S3 Table Different architectures of fully connected networks (number of neurons per layer × number of hidden layers). Best of at least three runs with different batch sizes with a constant train-validation-test split.
(PNG)

S4 Table Validation loss and test accuracy of prediction of the turning side of the focal fish after 1 second, changing variables in the aggregation subnetwork. 25 neighbours, attention network, mean of three runs. Elsewhere in this article, we use *v*, *v*_*i*_ and (*x*_*i*_, *y*_*i*_), the simplest to plot among the two sets of four variables with lower validation loss.
(PNG)

S5 Table Accuracy of the prediction of large turns for videos of different number of animals. Accuracy of the prediction for all turns and for large turns (20°-160°) for videos of different number of animals. 25 neighbours, average of three runs.
(PNG)
