# Supplementary figures and images for: Deep attention networks reveal the rules of collective motion in zebrafish
Source: PLoS Comput Biol. 2019 Sep 13;15(9):e1007354. doi: 10.1371/journal.pcbi.1007354 (PMC6760814; doi:10.1371/journal.pcbi.1007354)

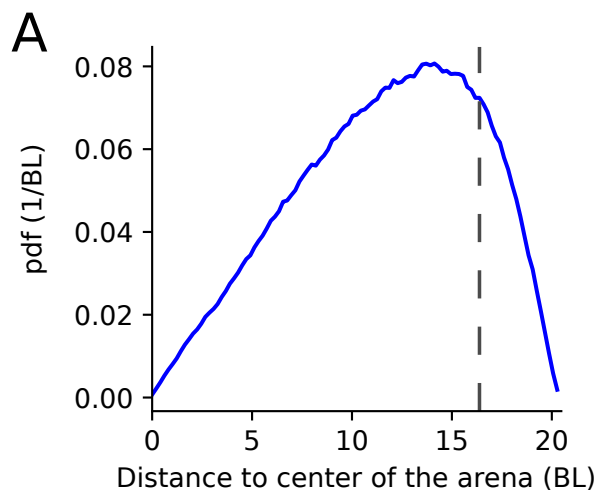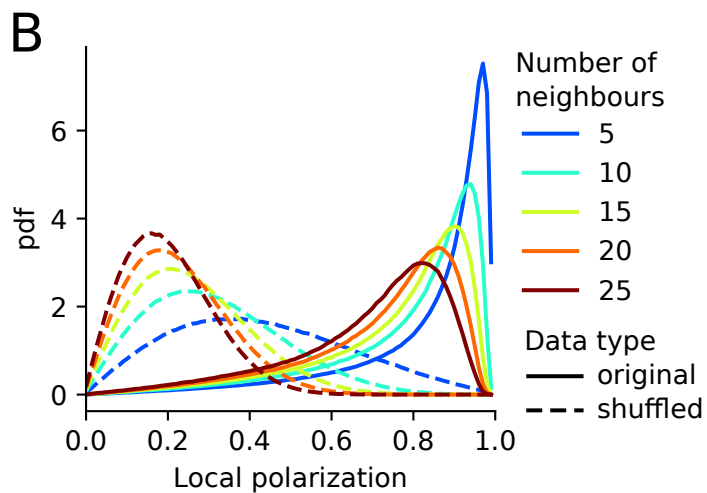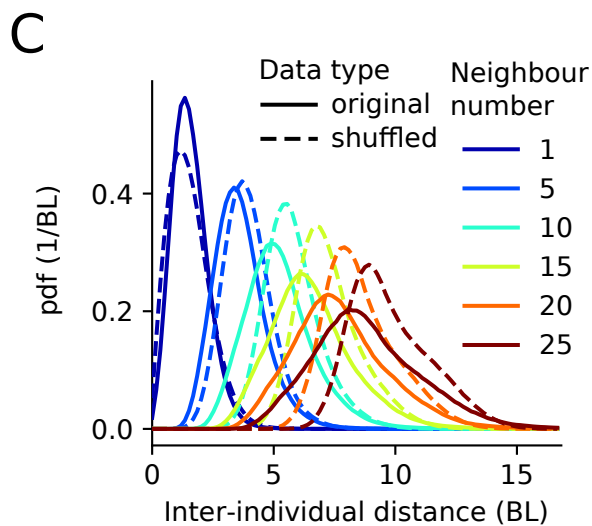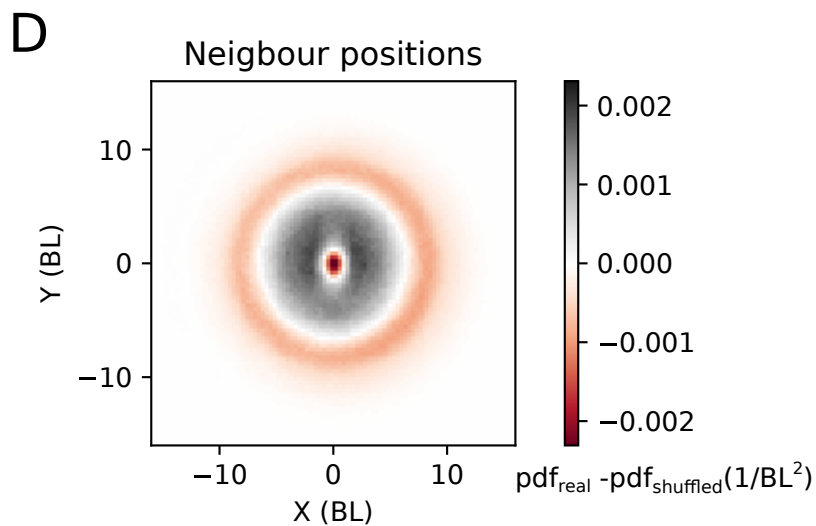

Supplement: S1 Fig — A Probability density function (pdf) of the distance to the center of the arena. The black vertical line marks the point 0.8 radius from the center; data to its right is neither used to train nor to evaluate the model to avoid direct border effects. B Polarisation, calculated locally in each frame for each focal fish and a different number of its closest neighbours, both for original and shuffled trajectories. C pdf of interindividual distances, in each frame for each fish to each of its closest neighbours. D Difference between the pdfs of relative locations of the 25 nearest neighbours in original and shuffled trajectories. (PDF) [file pcbi.1007354.s001.pdf]

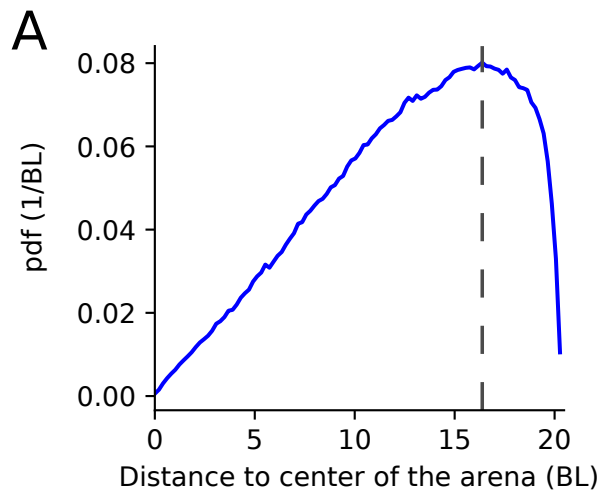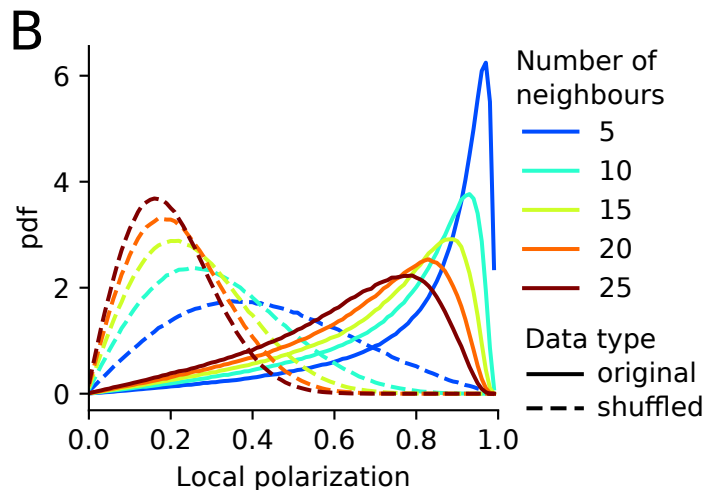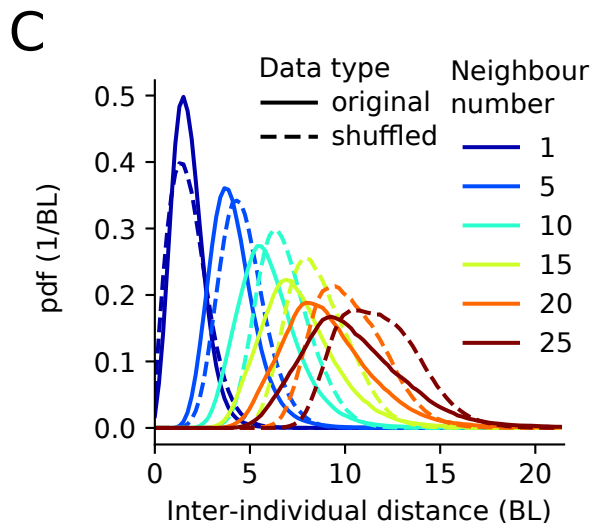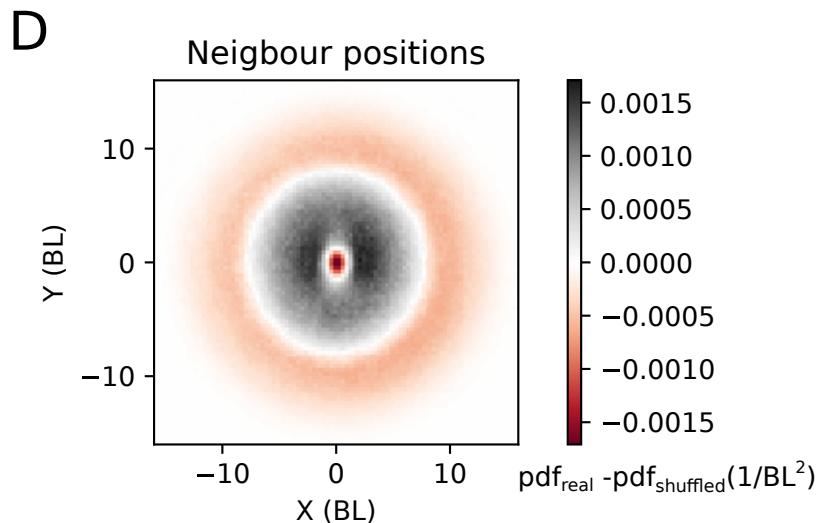

Supplement: S2 Fig — As in S1 Fig. (PDF) [file pcbi.1007354.s002.pdf]

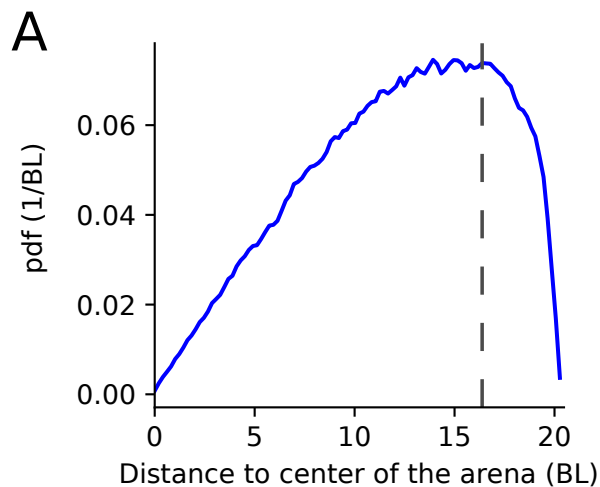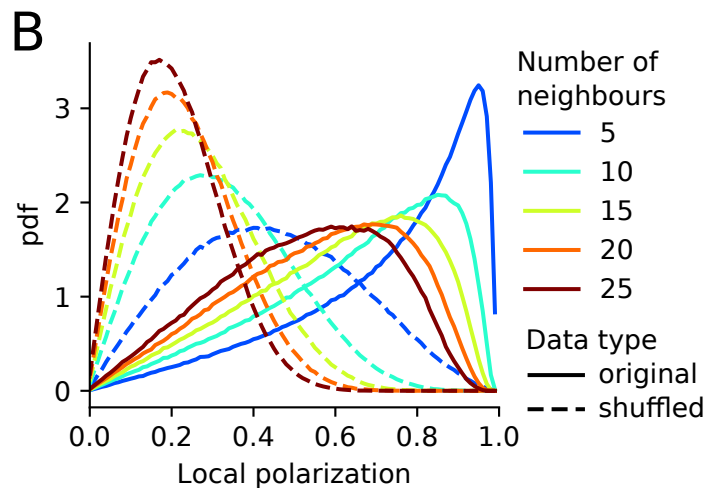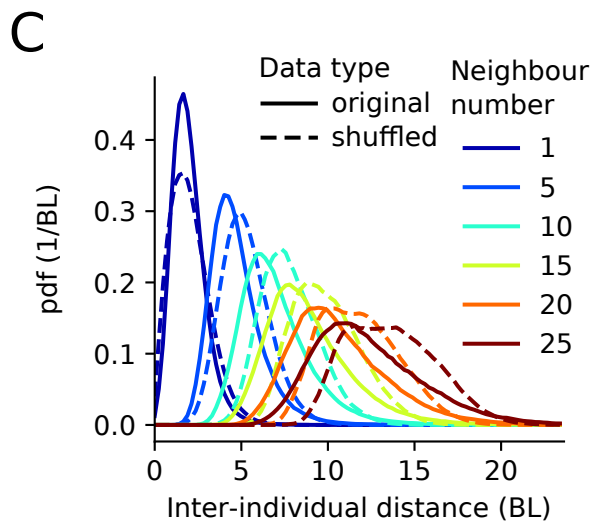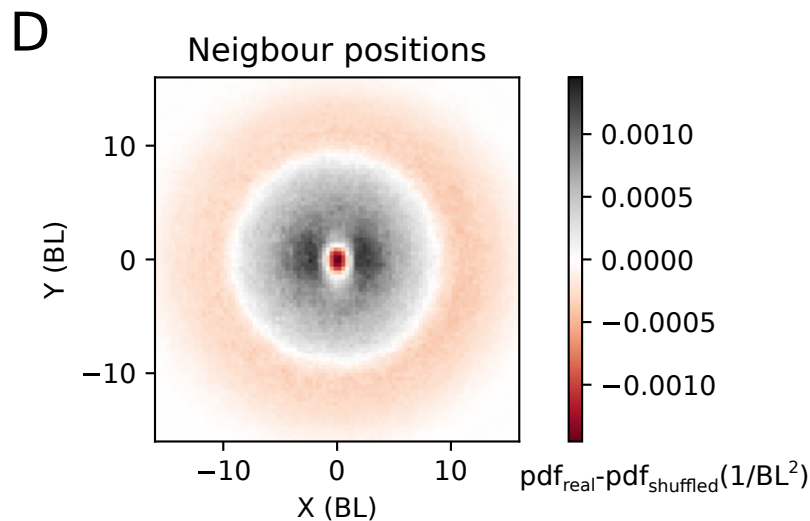

Supplement: S3 Fig — As in S1 Fig. (PDF) [file pcbi.1007354.s003.pdf]

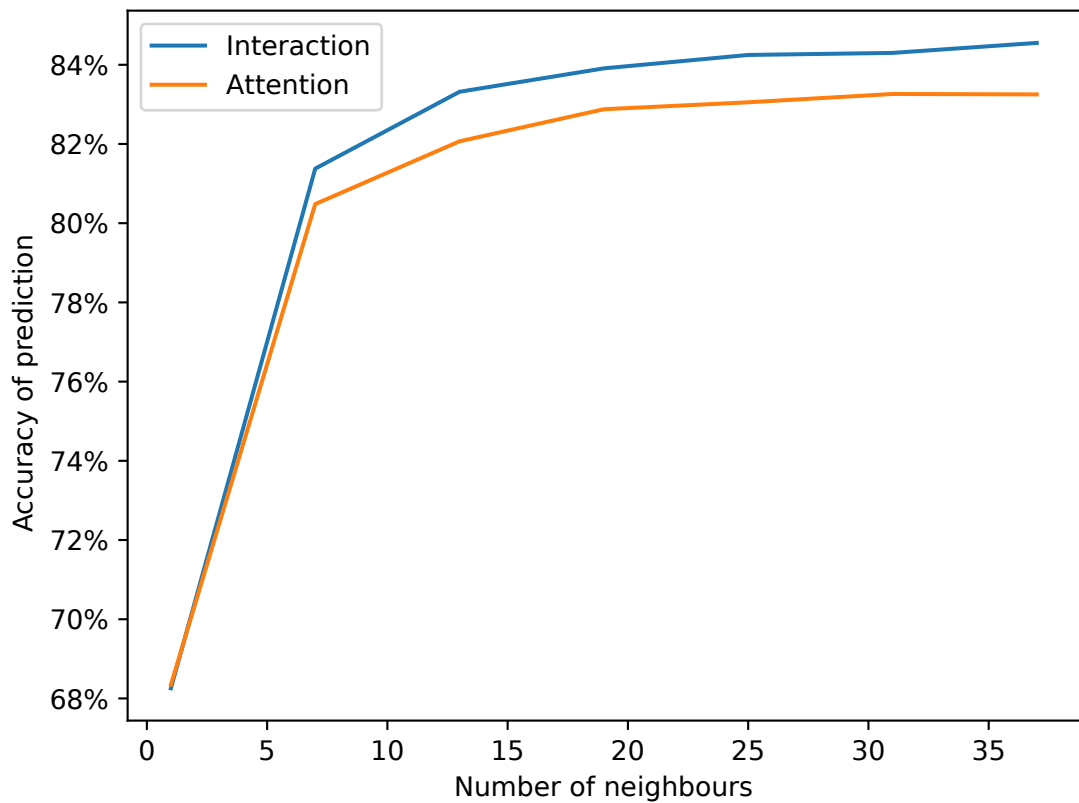

Supplement: S4 Fig — One run for each network/condition. Both the interaction network (blue) and the attention network (orange) improve in accuracy with the number of neighbours, and then plateau after approx. 20 neighbours. (PDF) [file pcbi.1007354.s004.pdf]

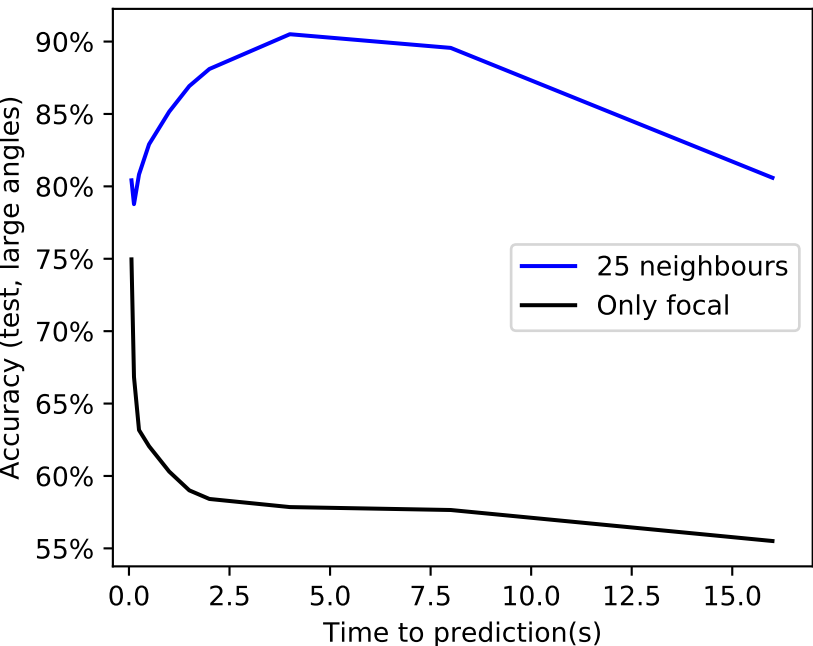

Supplement: S5 Fig — Mean of three runs, taking the test set at different positions of the video. The prediction from an aggregation model with 25 neighbours (blue) and from a model that is blind to any social information (black). Accuracy for immediate futures (less than 100 ms) is high for both models, because of correlations in the acceleration. Then it decreases for both models, but accuracy with 25 neighbours has a broad maximum when predicting futures between 1 and 10 s, and then slowly drops when predicting more distant futures. (PDF) [file pcbi.1007354.s005.pdf]

**A**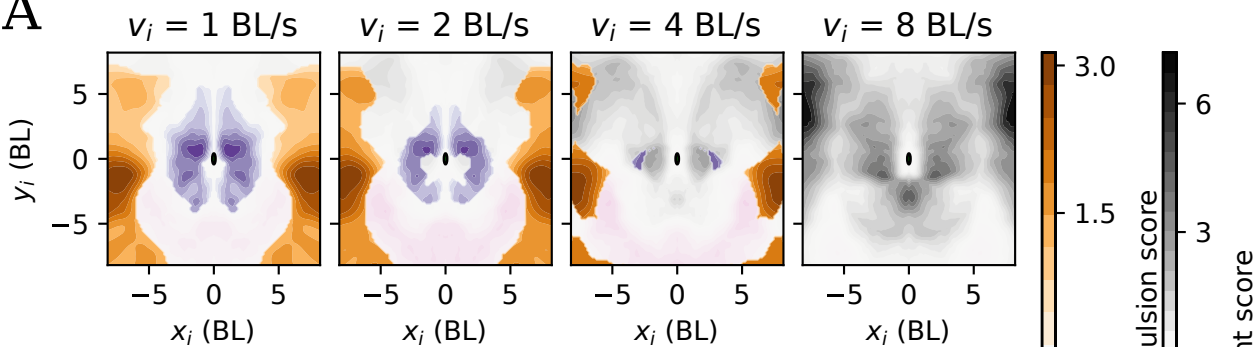**B**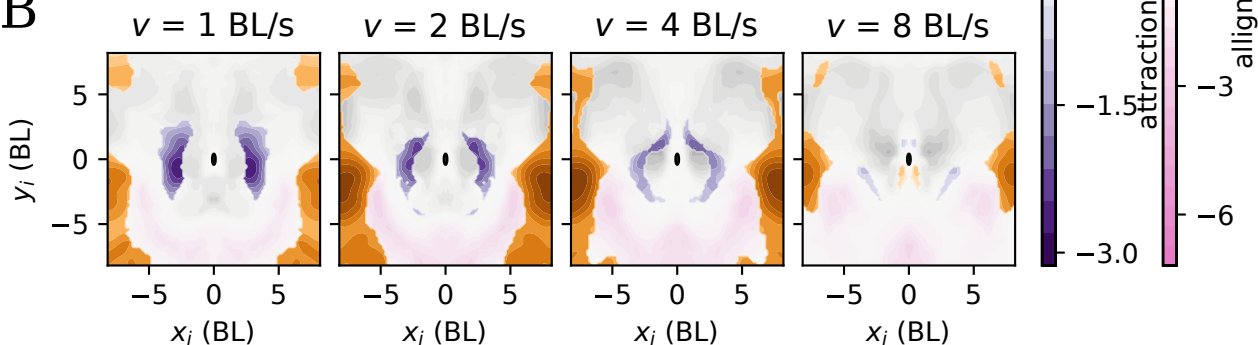**C**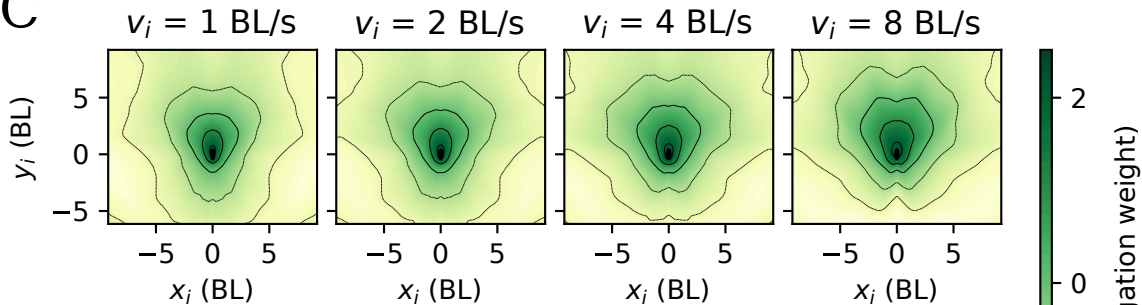**D**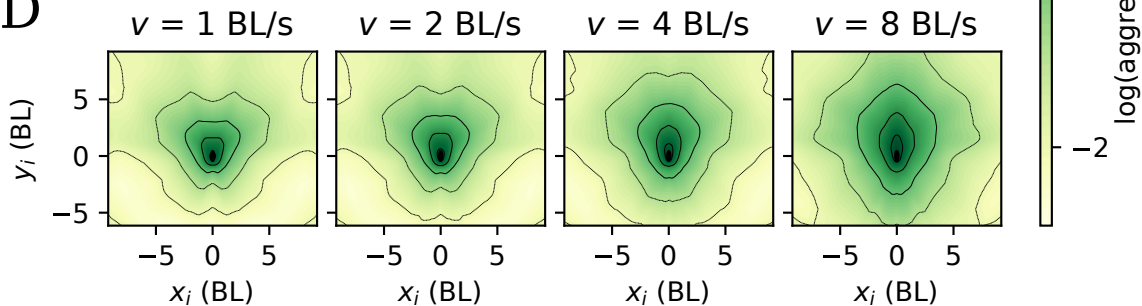

Supplement: S6 Fig — A Same as Fig 3A. B Same as Fig 3B C Same as Fig 5. Note how high-attention areas are closer to the focal fish. D Same as Fig 5. (PDF) [file pcbi.1007354.s006.pdf]

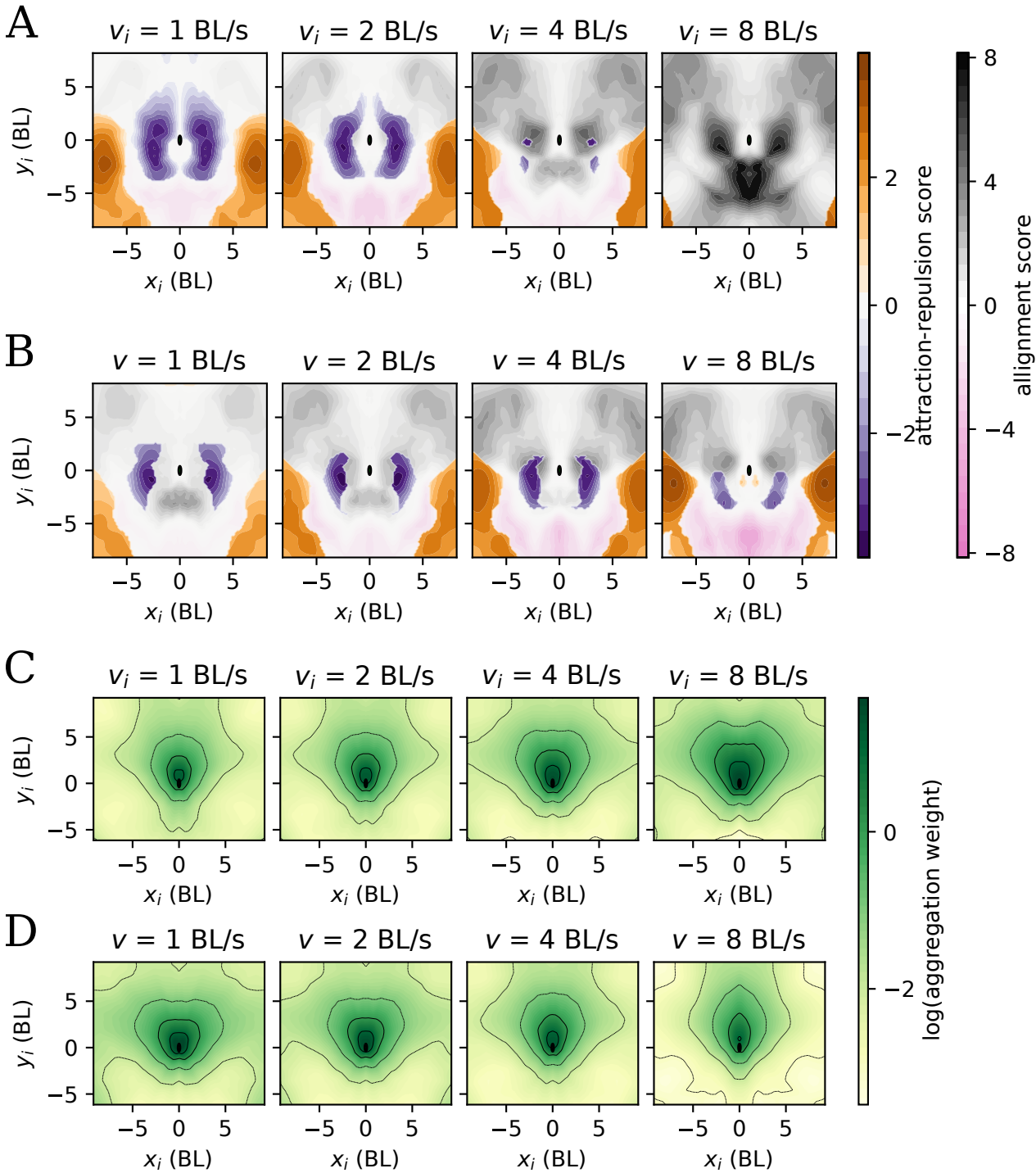

Supplement: S7 Fig — A Same as Fig 3A. B Same as Fig 3B C Same as Fig 5. D Same as Fig 5. (PDF) [file pcbi.1007354.s007.pdf]

**A**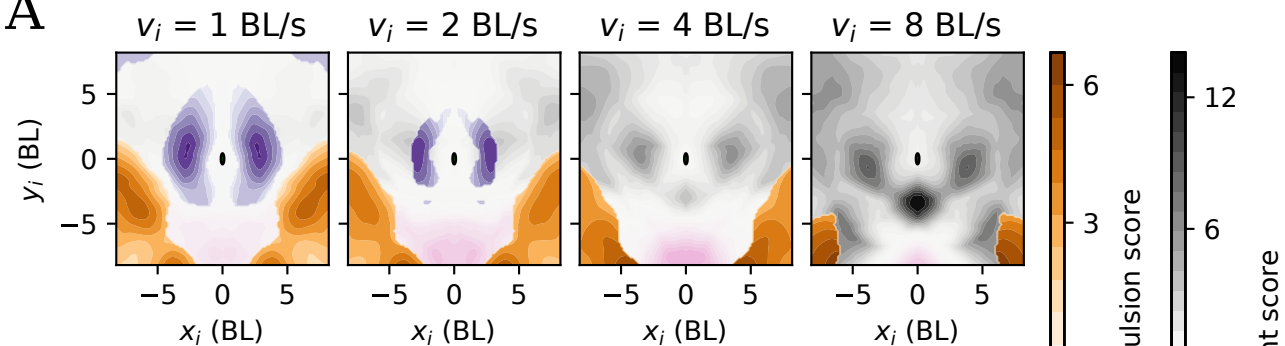**B**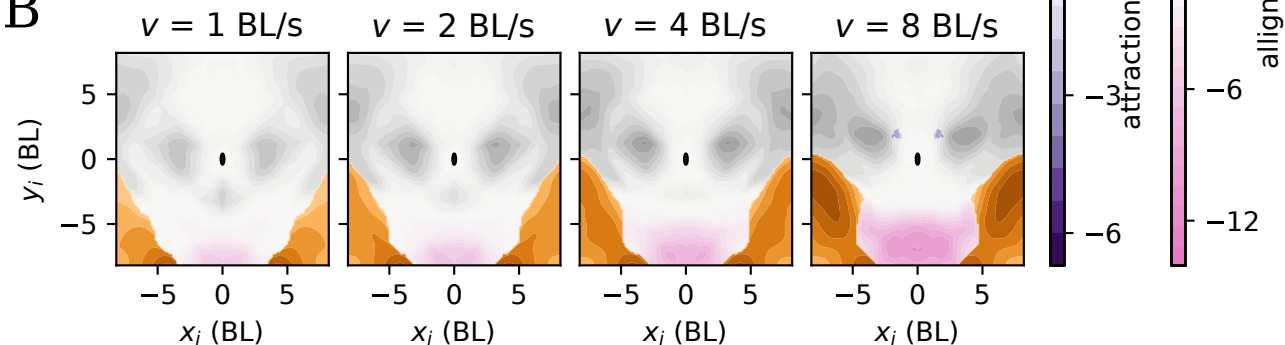**C**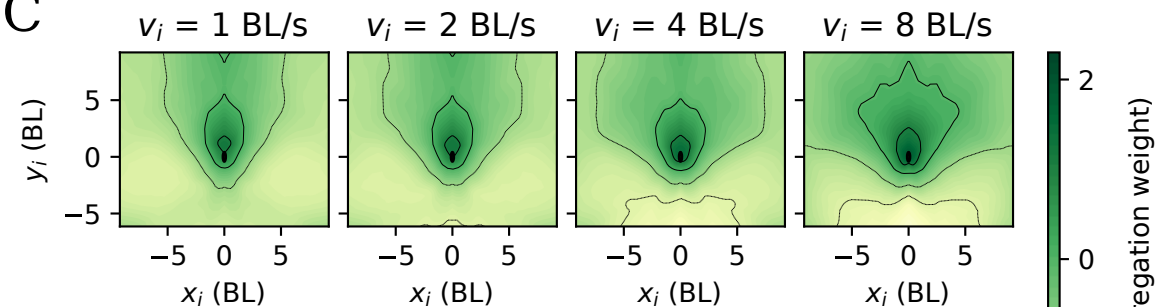**D**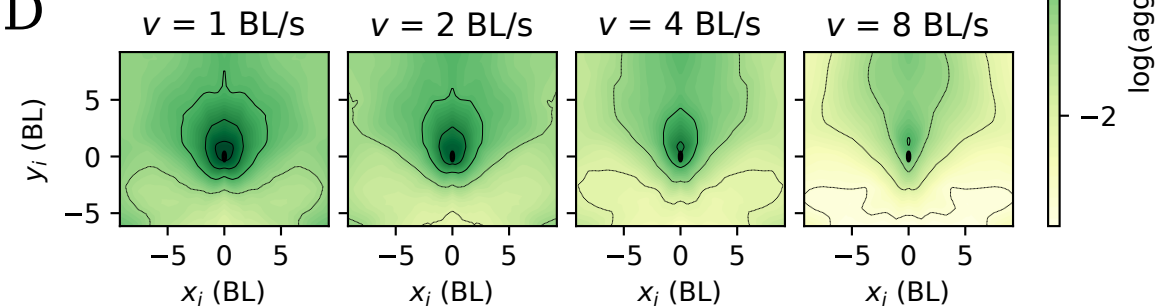

Supplement: S8 Fig — A Same as Fig 3A. B Same as Fig 3B C Same as Fig 5. Note how high-attention areas are closer to the front. D Same as Fig 5. (PDF) [file pcbi.1007354.s008.pdf]

**A** $v_i = 1$  BL/s $v_i = 2$  BL/s $v_i = 4$  BL/s $v_i = 8$  BL/s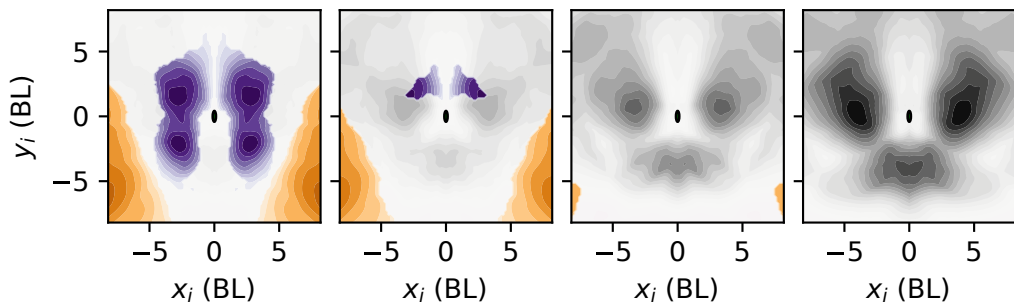**B** $v = 1$  BL/s $v = 2$  BL/s $v = 4$  BL/s $v = 8$  BL/s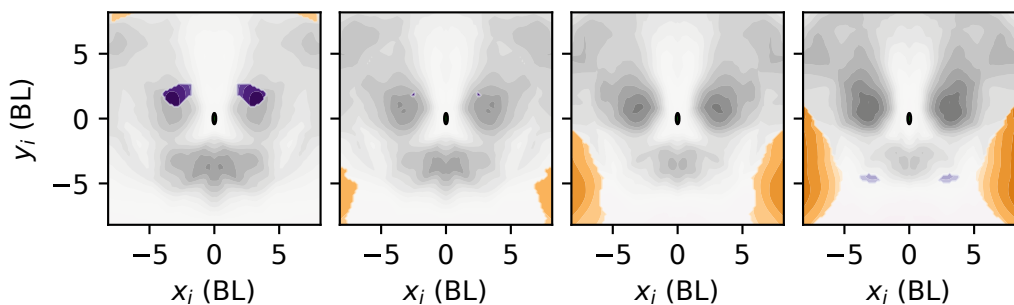**C** $v_i = 1$  BL/s $v_i = 2$  BL/s $v_i = 4$  BL/s $v_i = 8$  BL/s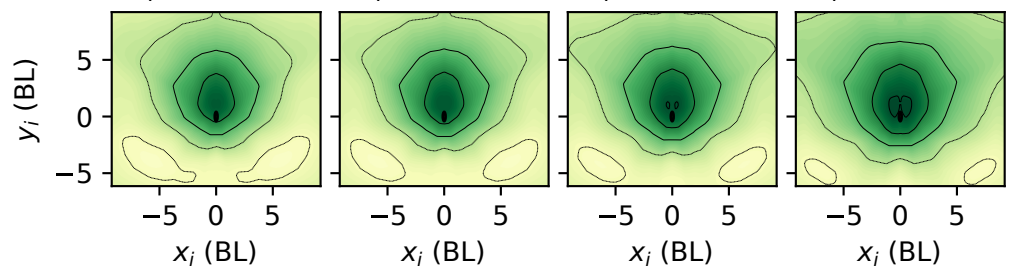**D** $v = 1$  BL/s $v = 2$  BL/s $v = 4$  BL/s $v = 8$  BL/s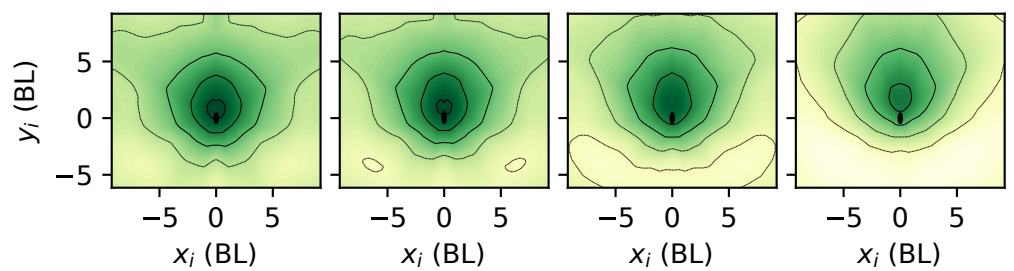

Supplement: S9 Fig — A Same as Fig 3A. The most conspicuous difference with Fig 3A is the weakening of anti-alignment B Same as Fig 3B C Same as Fig 5. D Same as Fig 5. Note the comparatively weak attention at the back of the focal. (PDF) [file pcbi.1007354.s009.pdf]

**A** $v_i = 1$  BL/s $v_i = 2$  BL/s $v_i = 4$  BL/s $v_i = 8$  BL/s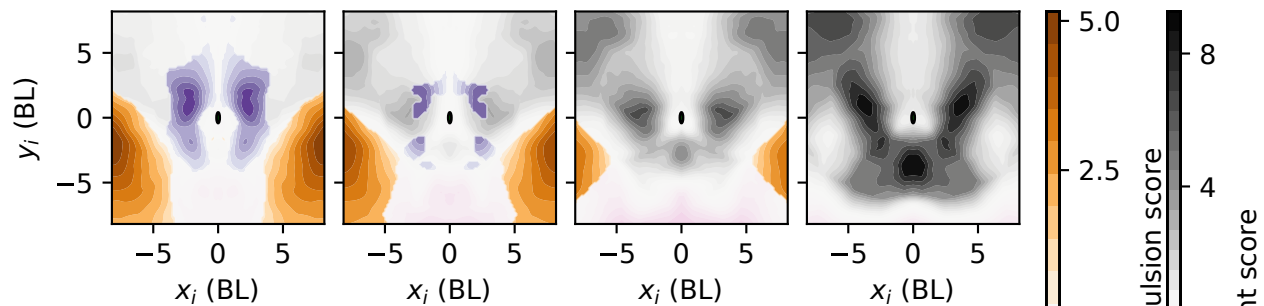**B** $v = 1$  BL/s $v = 2$  BL/s $v = 4$  BL/s $v = 8$  BL/s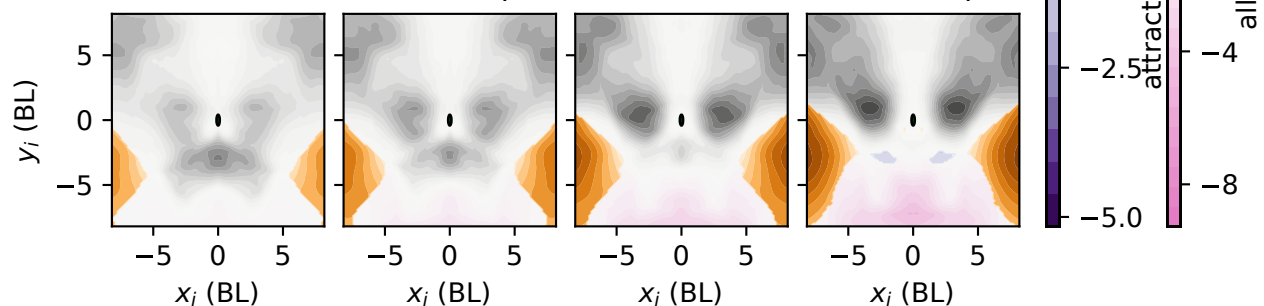**C** $v_i = 1$  BL/s $v_i = 2$  BL/s $v_i = 4$  BL/s $v_i = 8$  BL/s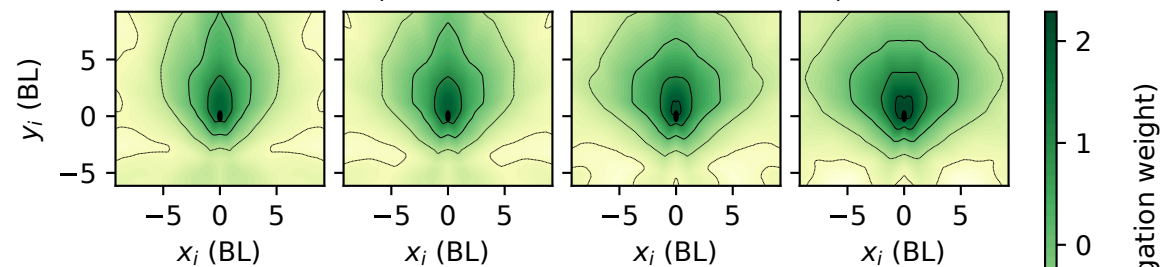**D** $v = 1$  BL/s $v = 2$  BL/s $v = 4$  BL/s $v = 8$  BL/s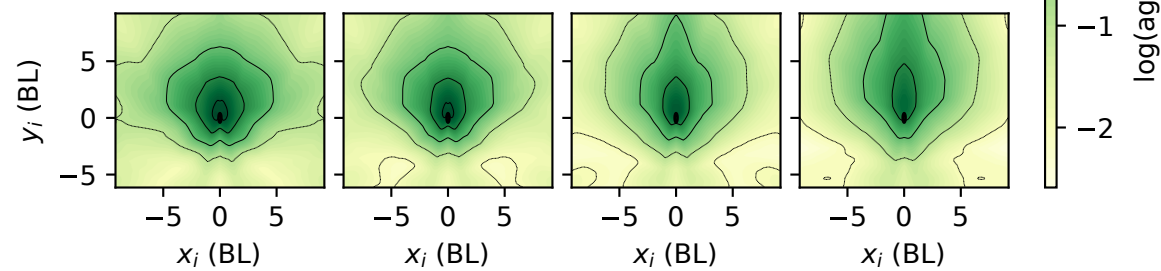

Supplement: S10 Fig — A Same as Fig 3A. The most conspicuous difference with Fig 3A is the weakening of anti-alignment B Same as Fig 3B C Same as Fig 5. D Same as Fig 5. (PDF) [file pcbi.1007354.s010.pdf]

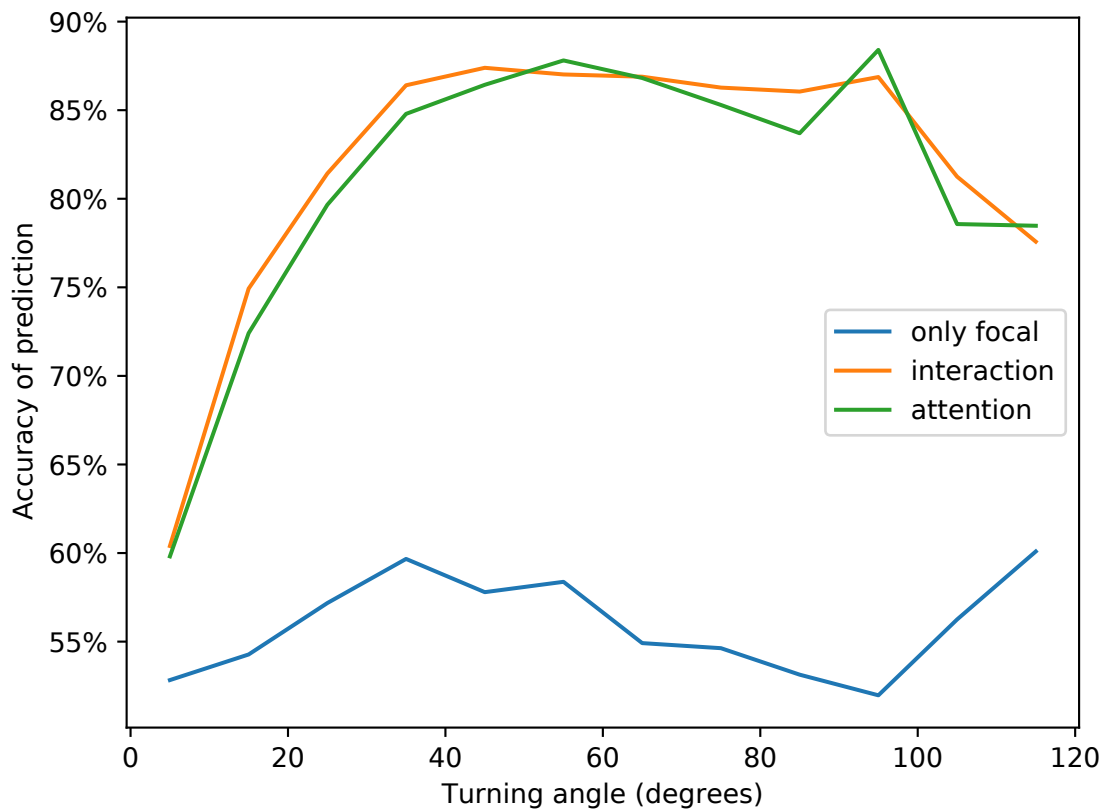

Supplement: S11 Fig — Data from one single run. When using only focal variables (blue) remains low at all turning angles. Both networks integrating information from 25 neighbours, interaction (orange) and attention (green) perform better at turning angles between 40 and 100. (PDF) [file pcbi.1007354.s011.pdf]

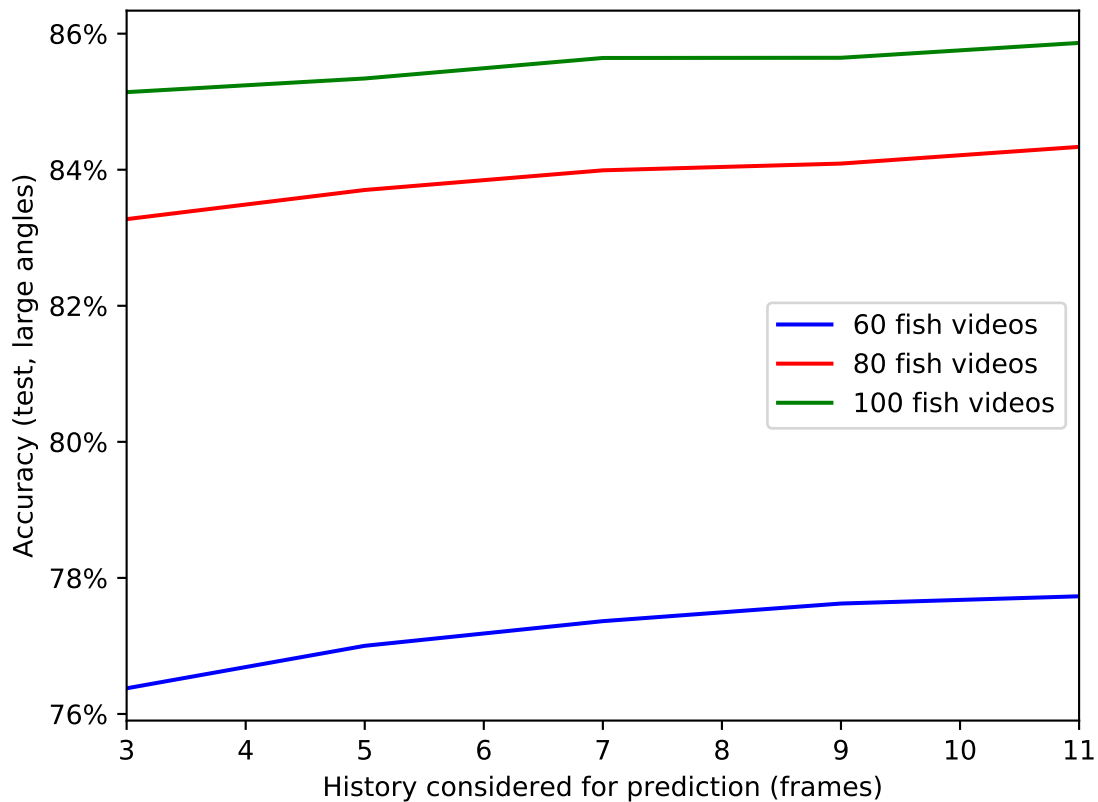

Supplement: S12 Fig — Mean of three runs, taking the test set at different positions in the video. Prediction accuracies increase when information from more frames in the past is available to the network. (PDF) [file pcbi.1007354.s012.pdf]

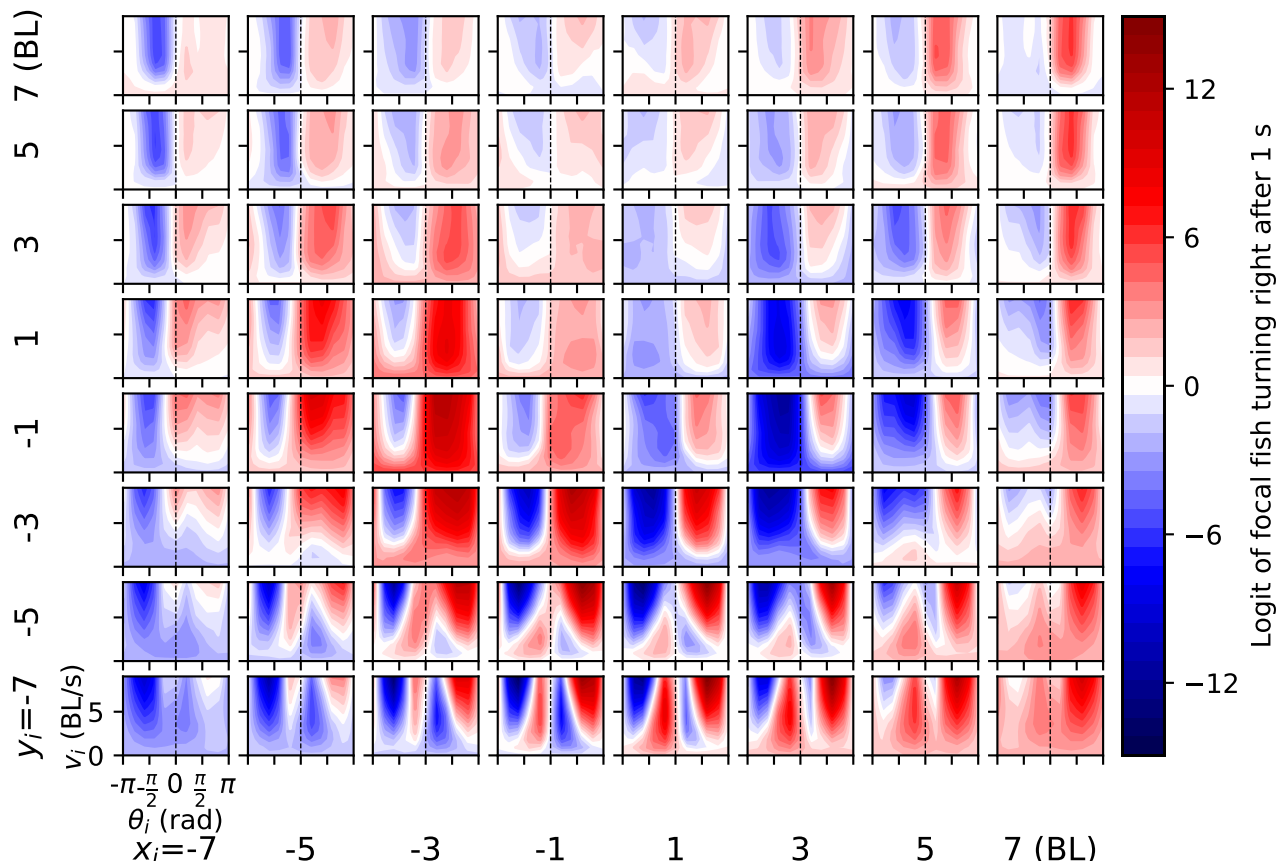

Supplement: S13 Fig — (PDF) [file pcbi.1007354.s013.pdf]

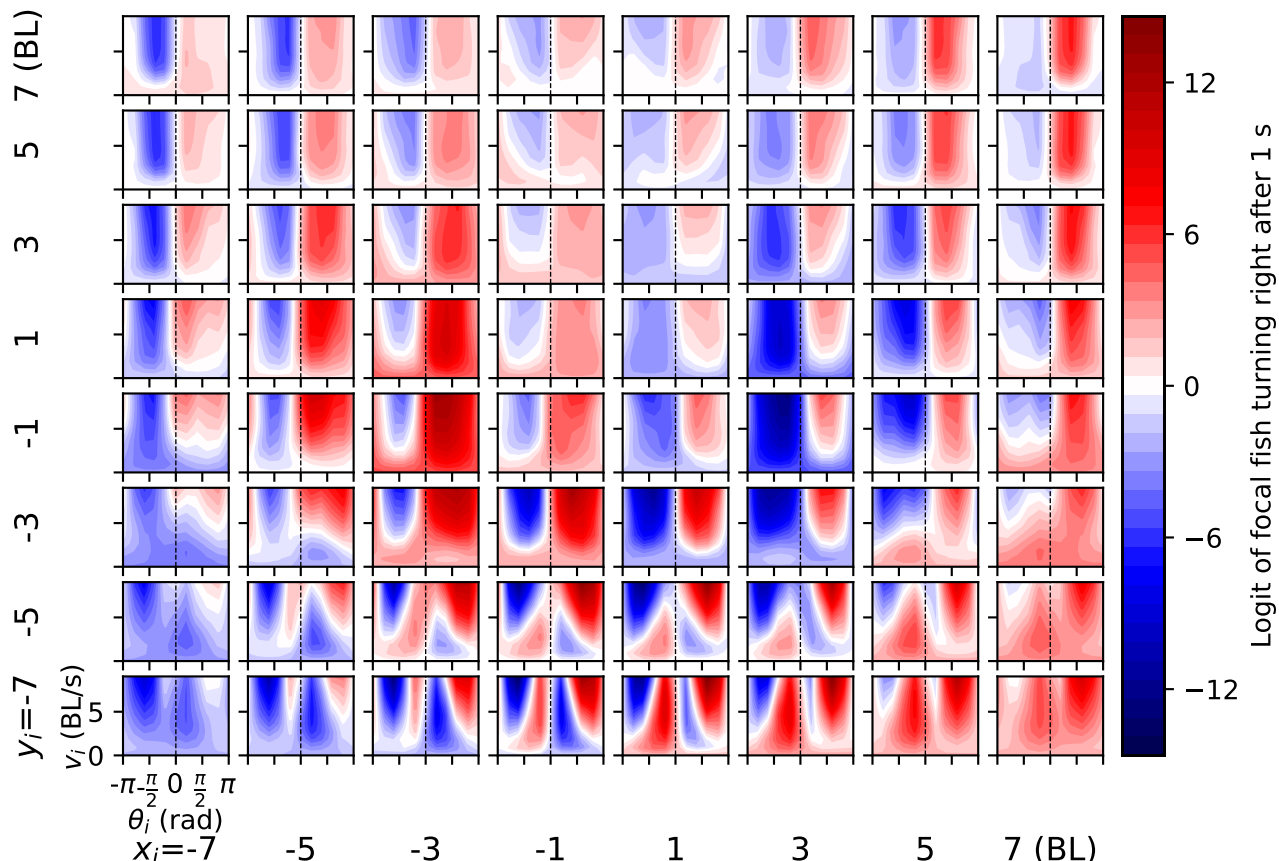

Supplement: S14 Fig — (PDF) [file pcbi.1007354.s014.pdf]

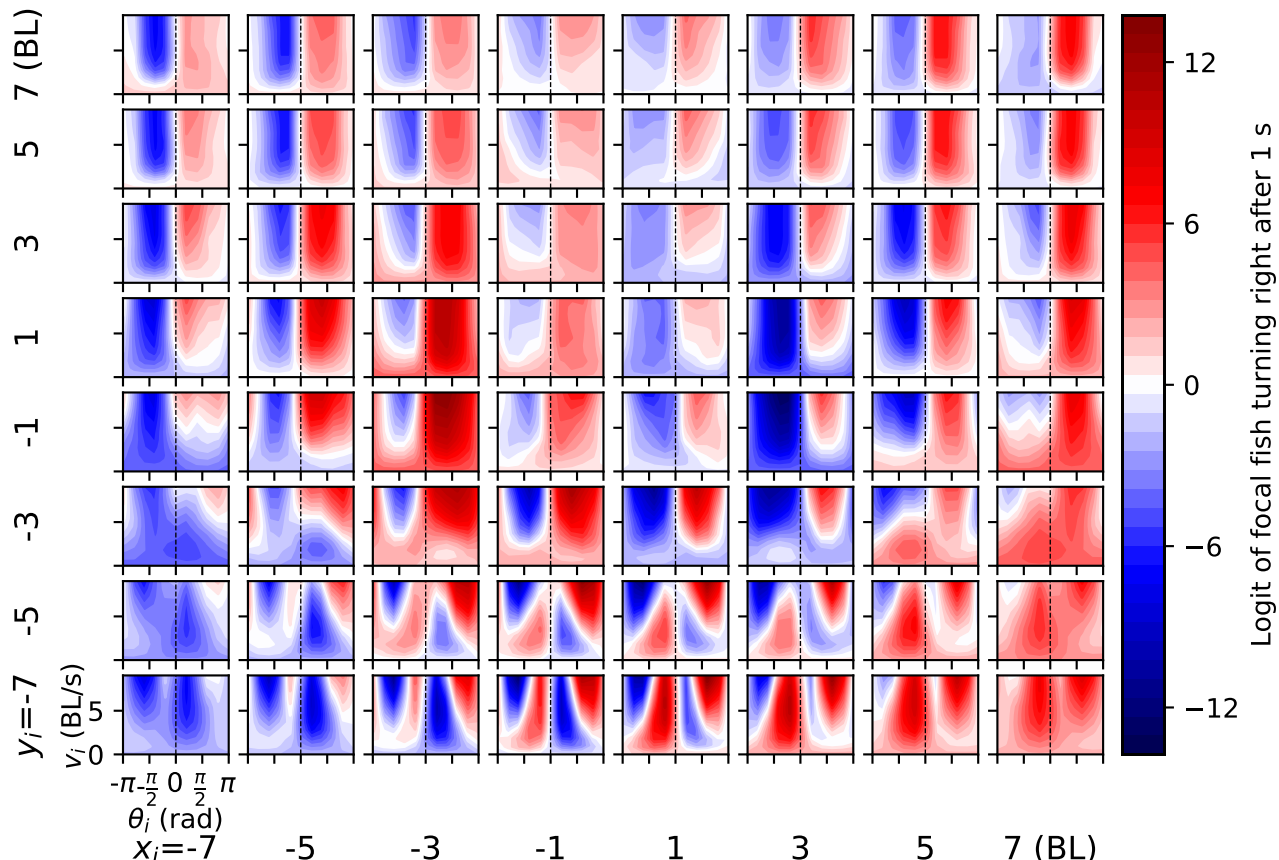

Supplement: S15 Fig — (PDF) [file pcbi.1007354.s015.pdf]

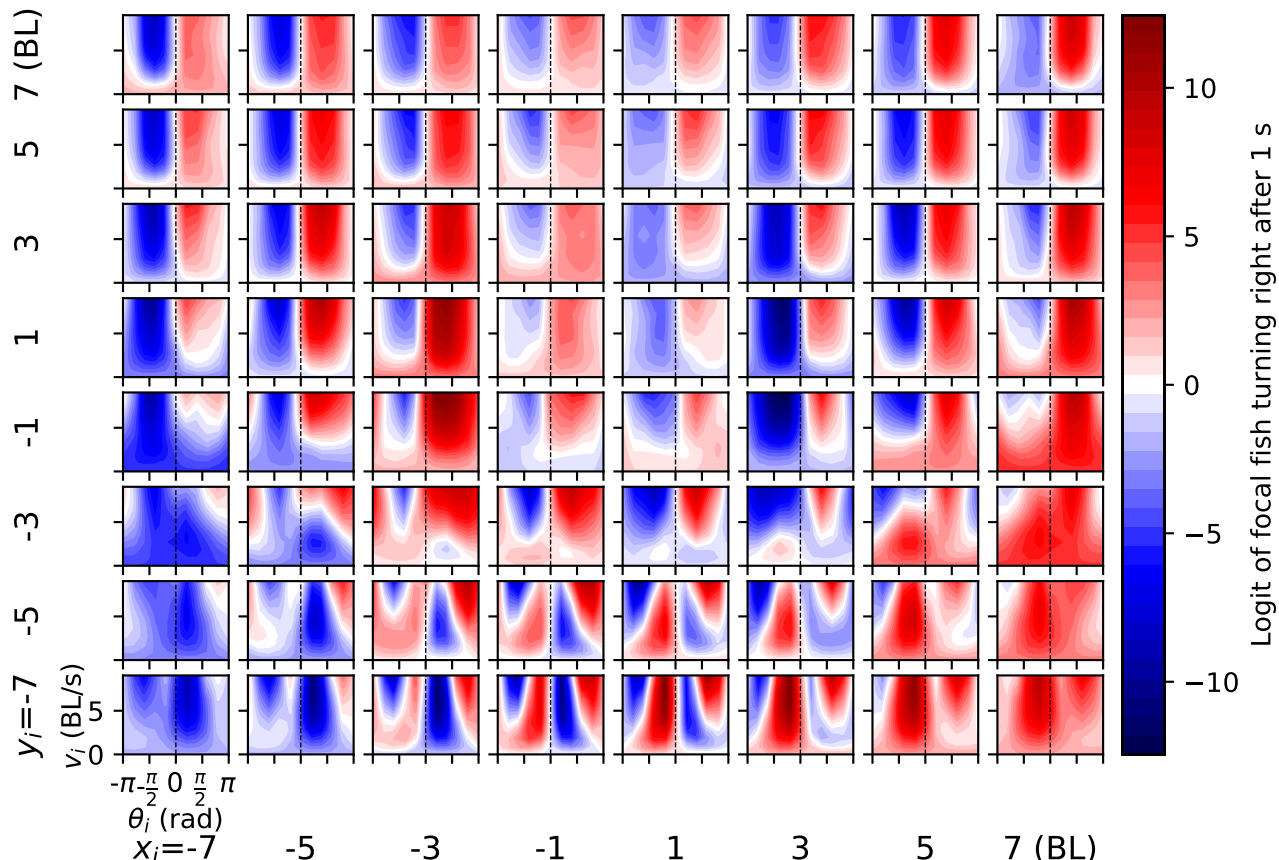

Supplement: S16 Fig — (PDF) [file pcbi.1007354.s016.pdf]

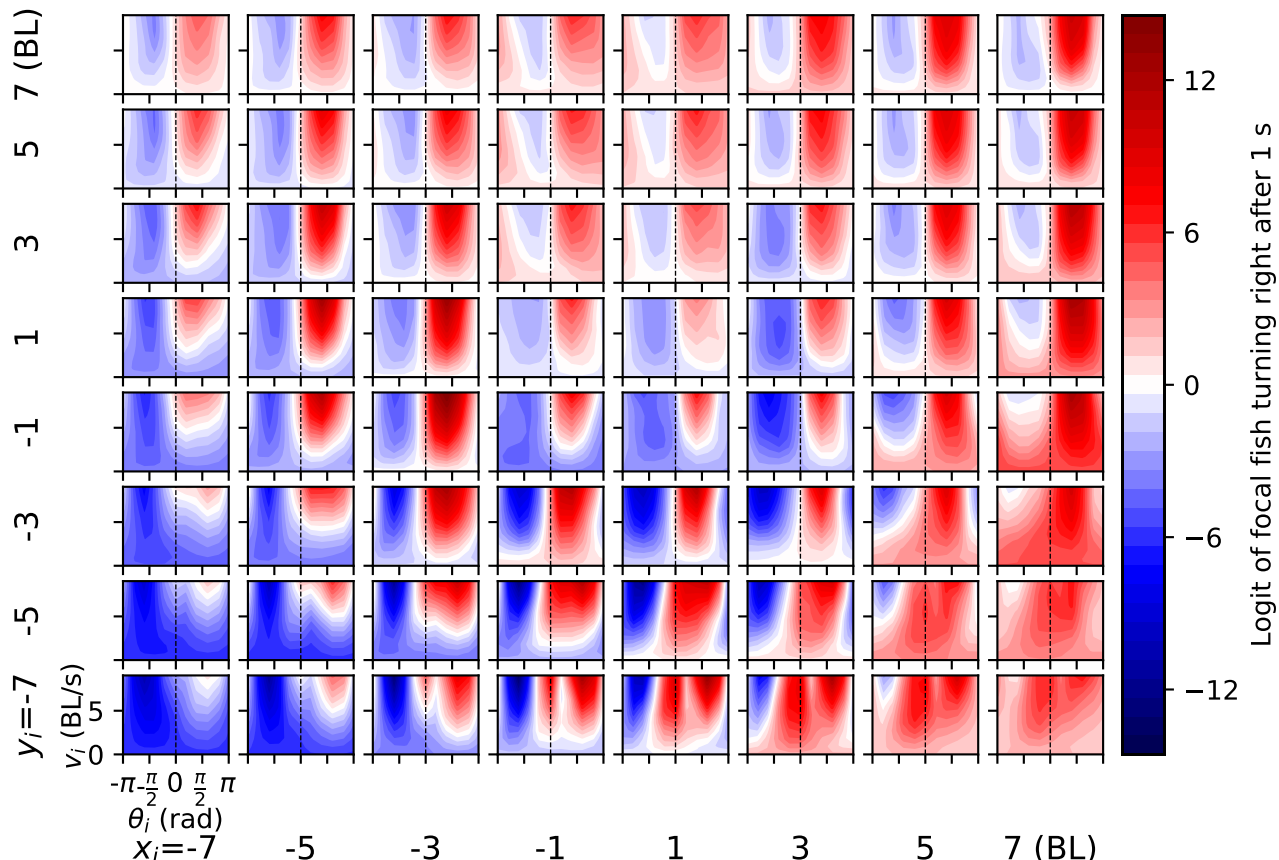

Supplement: S17 Fig — Focal normal acceleration fixed to a⊥ = 100 BL/s2. (PDF) [file pcbi.1007354.s017.pdf]

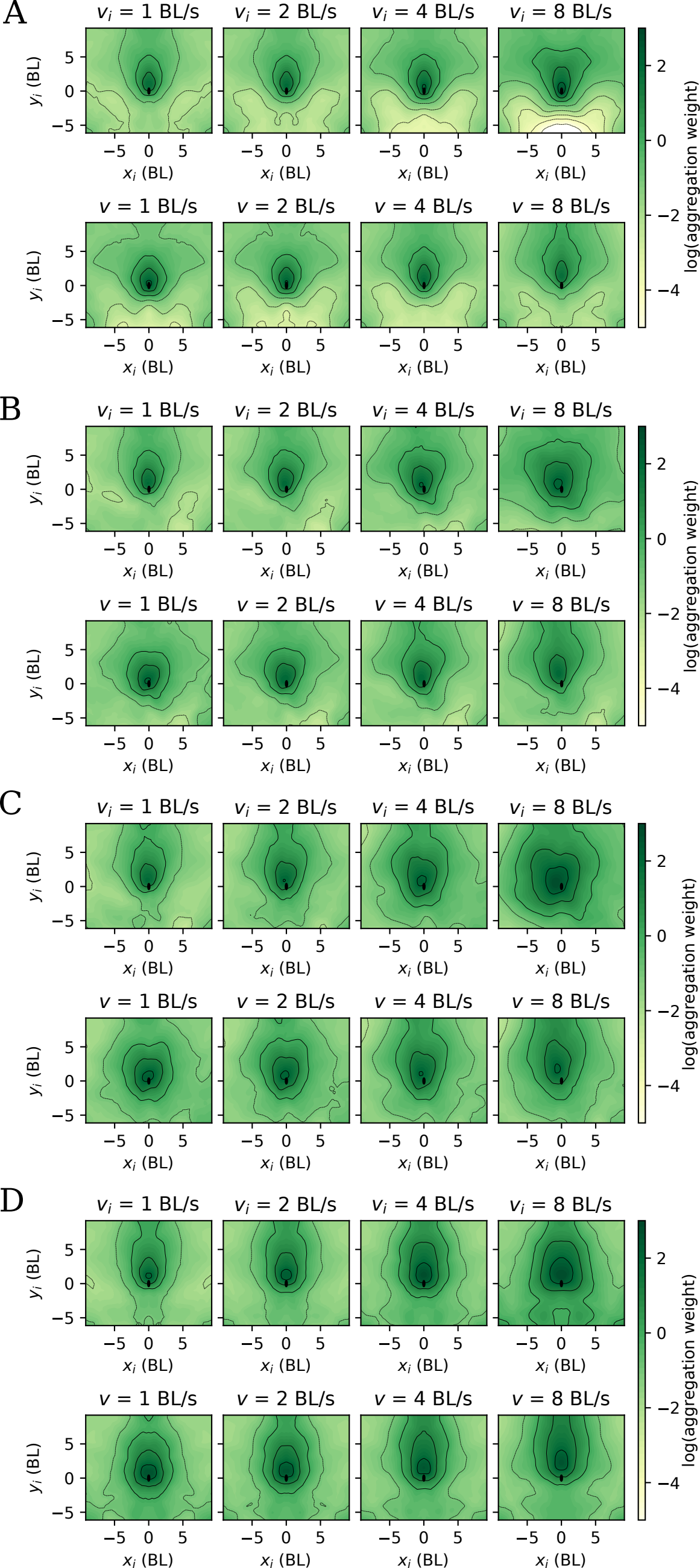

Supplement: S18 Fig — We show results for the network trained with the original data and the data shuffled according to Methods and Materials. Focal and neighbour speeds fixed to the median. (PDF) [file pcbi.1007354.s018.pdf]

Original data

Shuffled data

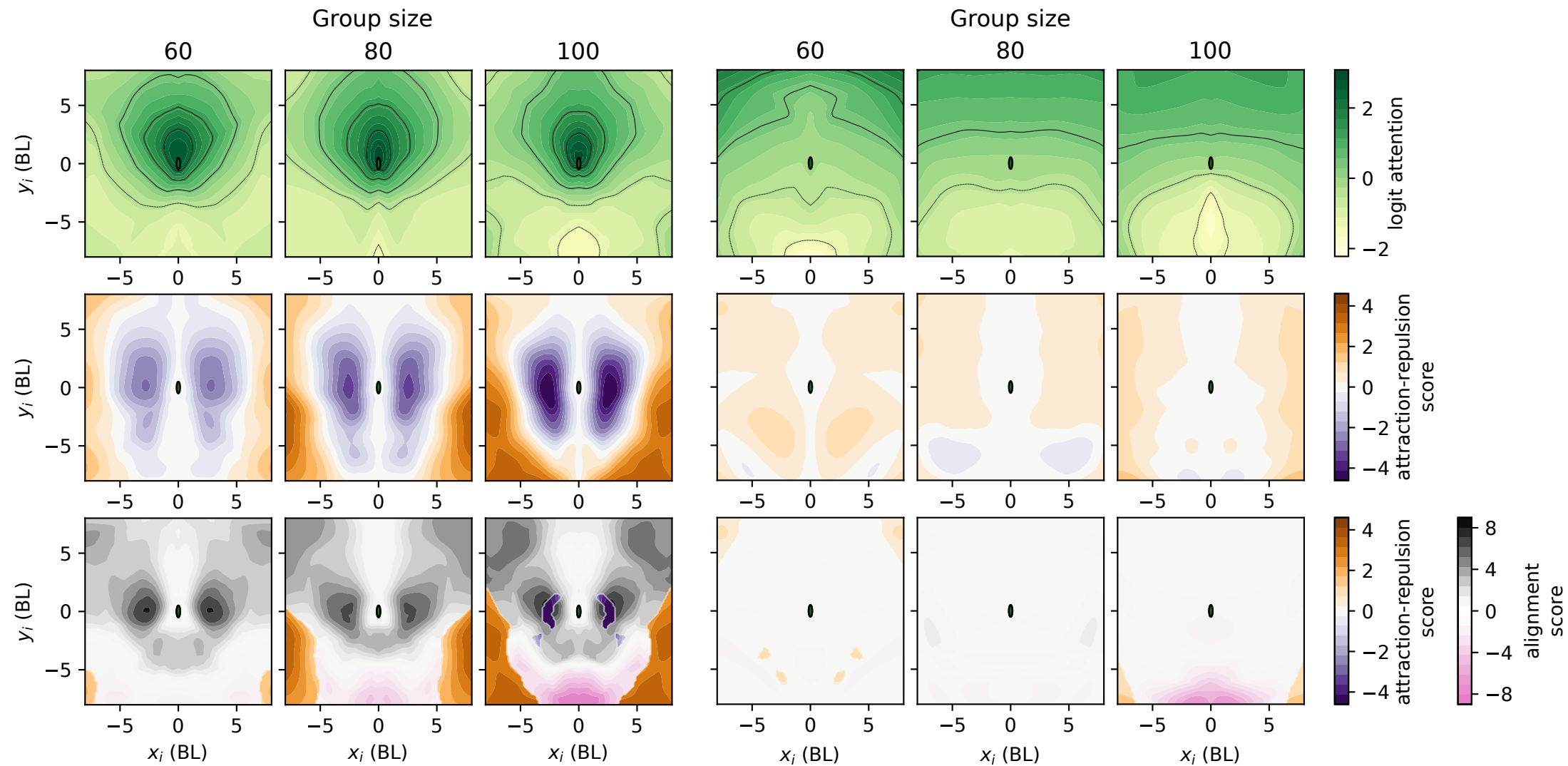

Supplement: S19 Fig — Same as Fig 5, but when the aggregation subnetwork is trained with the relative orientation of the neighbour, in addition to the variables used in the main text. A The neighbour is parallel (at 0 degrees) to the focal. B The neighbour is at 45 degrees (towards the right) with the focal, C the neighbour is perpendicular (90 degrees) and pointing to the right of the focal. D The neighbour is antiparallel (180 degrees) to the focal. (PDF) [file pcbi.1007354.s019.pdf]

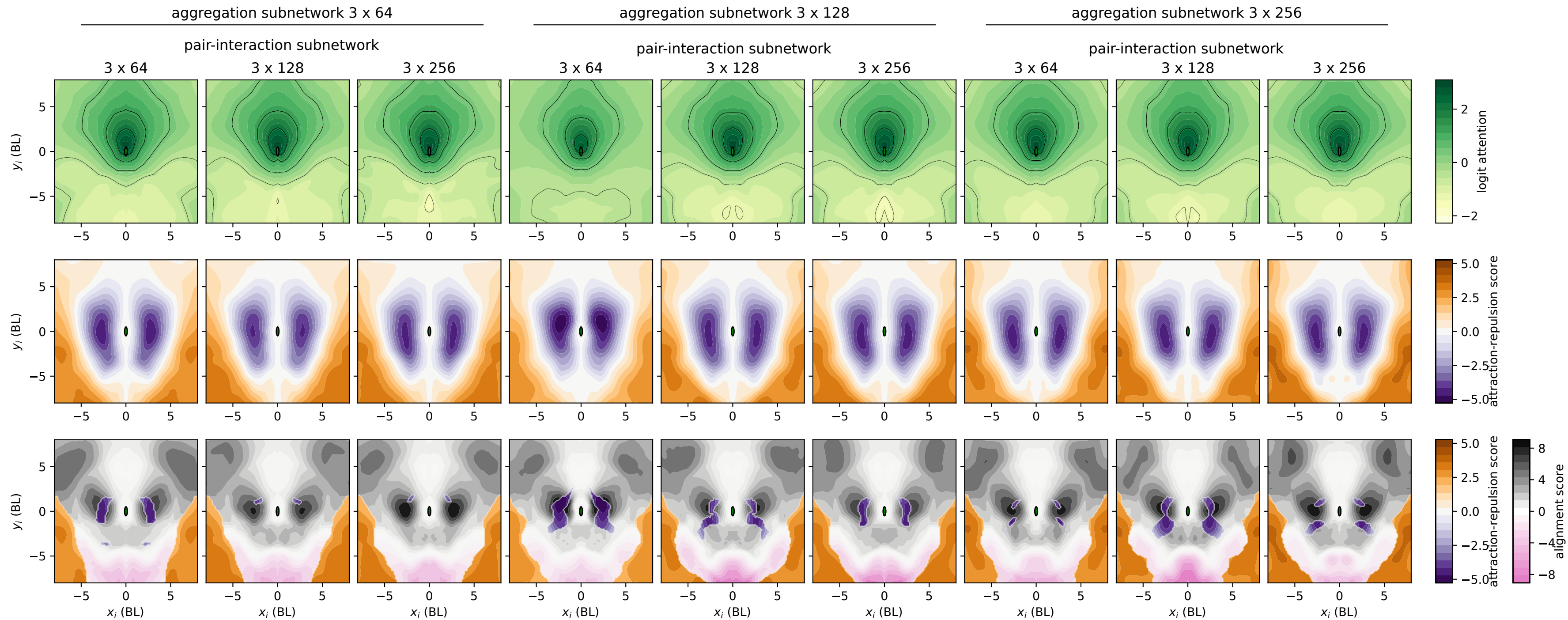

Supplement: S21 Fig — We show results for different number of neurons in the layers of both, the pair-wise interaction subnetwork, and the aggregation subnetwork. Focal and neighbour speeds fixed to the median. (PDF) [file pcbi.1007354.s021.pdf]

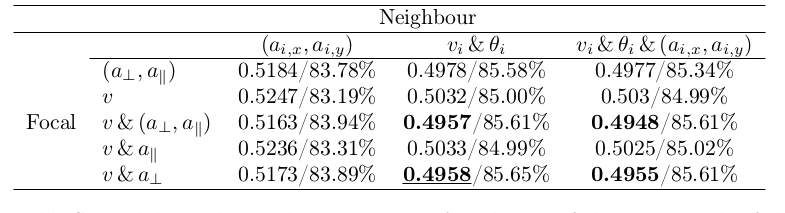

Supplement: S1 Table — 25 neighbours, interaction network, mean of three runs. In all cases, in addition to the variables mentioned in the table, we provide the relative position of the neighbour (xi, yi). Elsewhere in this article, we use v, a⊥, vi, θi and (xi, yi), the simplest among the sets of variables with high accuracy. Average of three runs with different train-validation-test splits. (PNG) [file pcbi.1007354.s022.png]

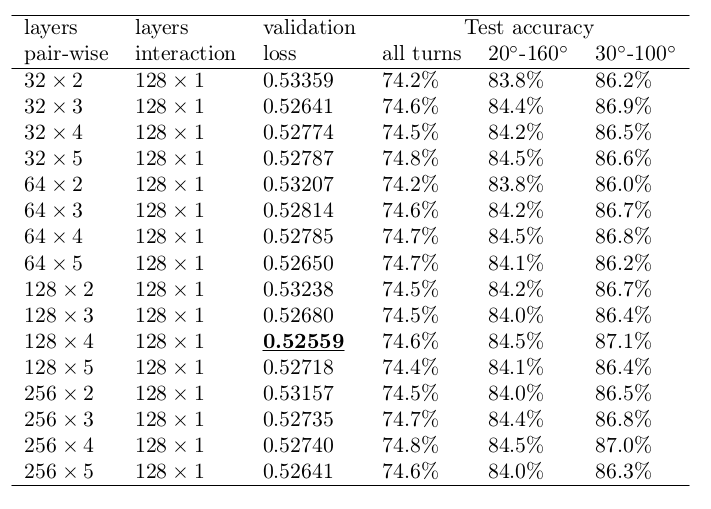

Supplement: S2 Table — Best (i.e. lowest validation loss) of at least three runs with different batch sizes, with a constant train-validation-test split. (PNG) [file pcbi.1007354.s023.png]

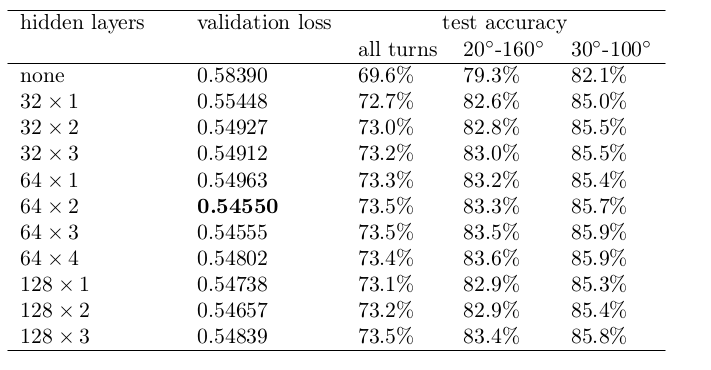

Supplement: S3 Table — Best of at least three runs with different batch sizes with a constant train-validation-test split. (PNG) [file pcbi.1007354.s024.png]

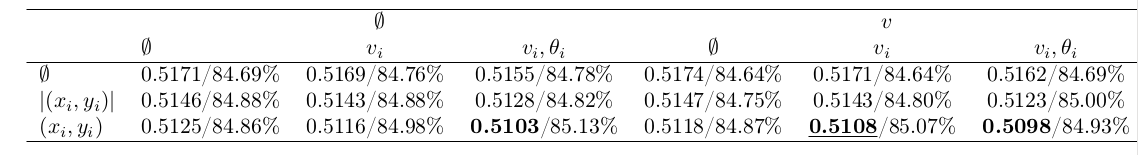

Supplement: S4 Table — 25 neighbours, attention network, mean of three runs. Elsewhere in this article, we use v, vi and (xi, yi), the simplest to plot among the two sets of four variables with lower validation loss. (PNG) [file pcbi.1007354.s025.png]

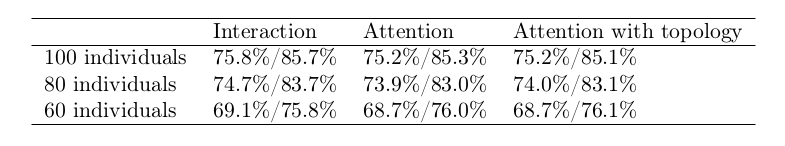

Supplement: S5 Table — Accuracy of the prediction for all turns and for large turns (20°-160°) for videos of different number of animals. 25 neighbours, average of three runs. (PNG) [file pcbi.1007354.s026.png]
