# Supplementary material for: Deep attention networks reveal the rules of collective motion in zebrafish
Source: PLoS Comput Biol. 2019 Sep 13;15(9):e1007354. doi: 10.1371/journal.pcbi.1007354 (PMC6760814; doi:10.1371/journal.pcbi.1007354)
Supplement: S20 Fig — We show results for different number of layers in both, the pair-wise interaction subnetwork, and the aggregation subnetwork. Focal and neighbour speeds fixed to the median. (PDF) [file pcbi.1007354.s020.pdf]

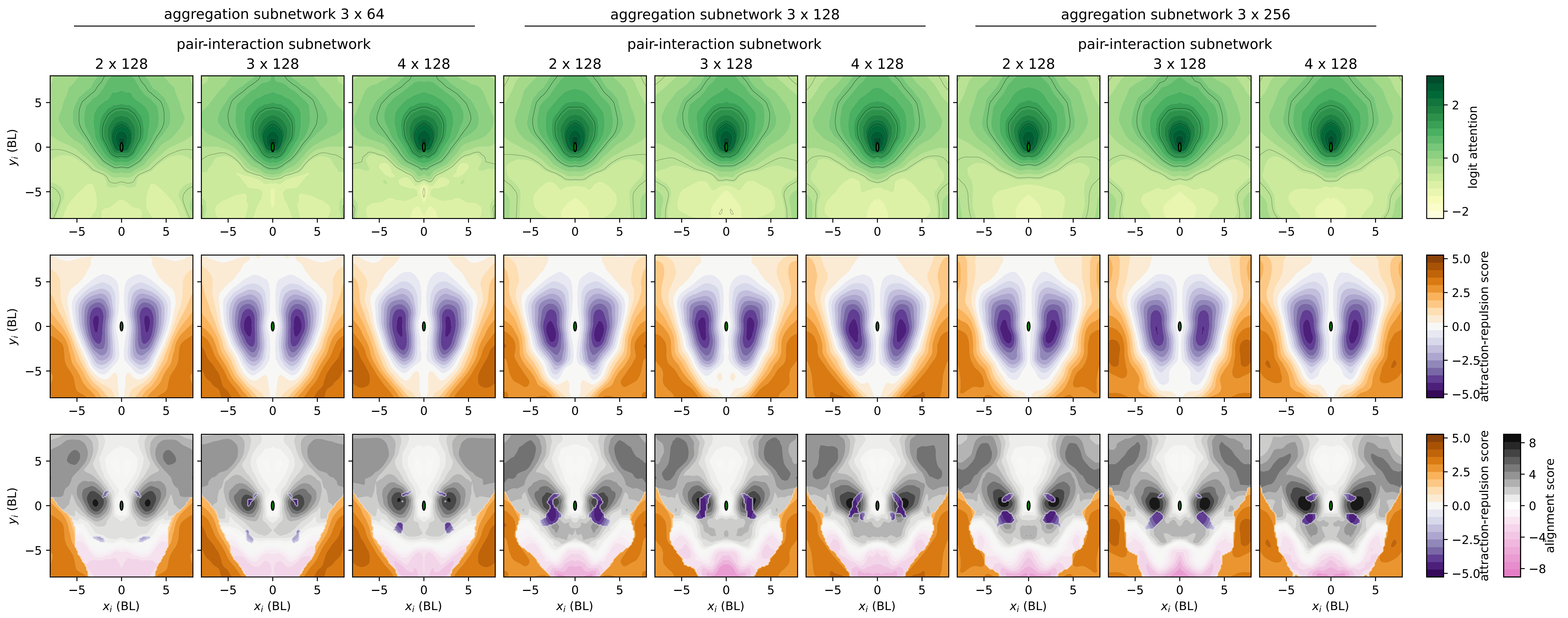

aggregation subnetwork 3 x 64
aggregation subnetwork 3 x 128
aggregation subnetwork 3 x 256
pair-interaction subnetwork
2 x 128
3 x 128
4 x 128
y_i (BL)
x_i (BL)
logit attention
attraction-repulsion score
alignment score
